# Supplementary material for: Activation of ARP2/3 and HSP70 Expression by Lipoteichoic Acid: Potential Bidirectional Regulation of Apoptosis in a Mastitis Inflammation Model
Source: Biomolecules. 2024 Jul 25;14(8):901. doi: 10.3390/biom14080901 (PMC11352453; doi:10.3390/biom14080901)
Supplement: Supplementary file 1 [file biomolecules-14-00901-s001.zip › Original Images of Western Blot.pptx]

## Slide 1
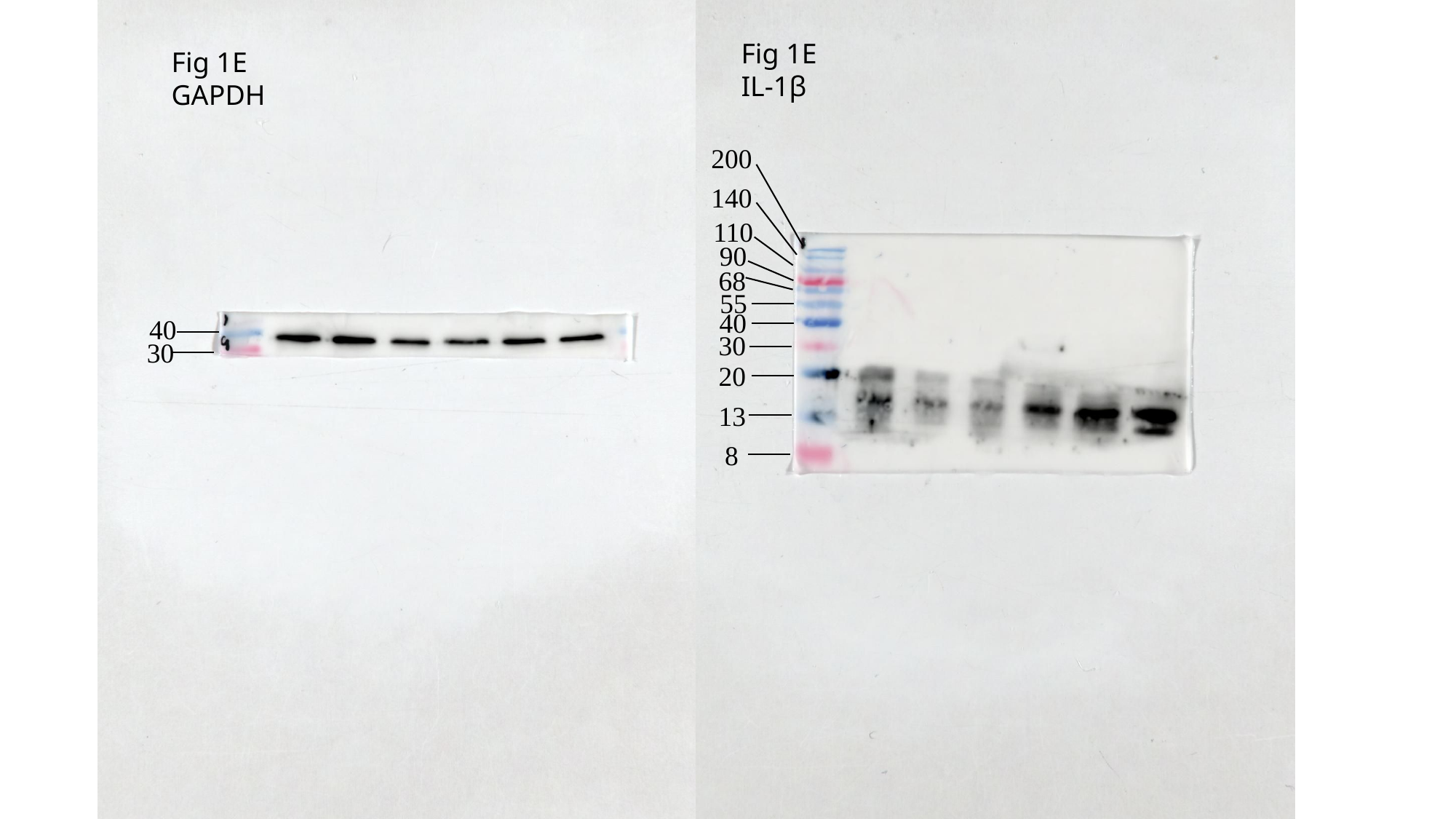

Fig 1E
IL-1β
Fig 1E GAPDH
200
140
110
90
68
55
40
40
30
30
20
13
8

## Slide 2
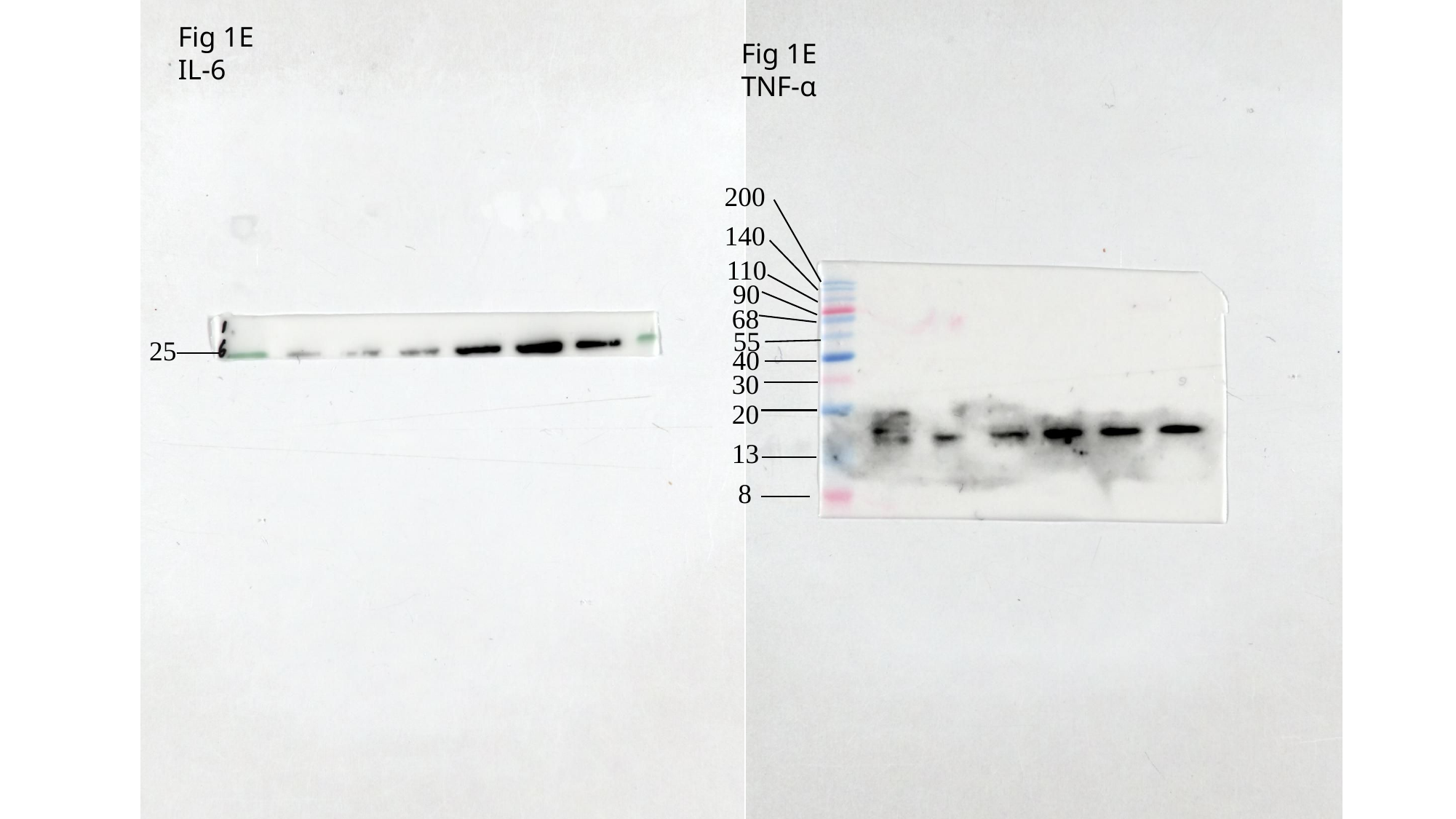

Fig 1E
IL-6
Fig 1E
TNF-α
200
140
110
90
68
55
25
40
30
20
13
8

## Slide 3
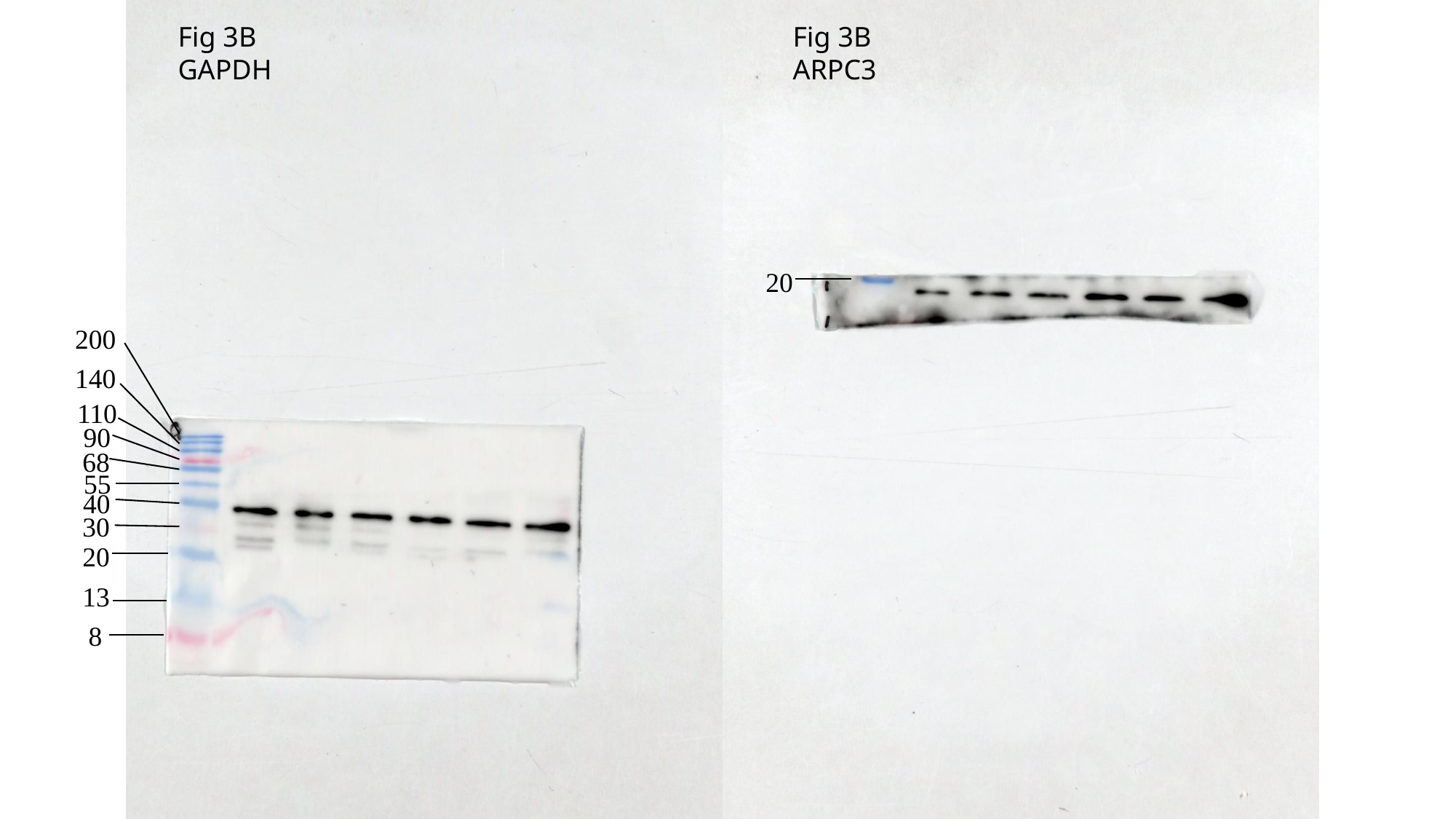

Fig 3B
GAPDH
Fig 3B
ARPC3
20
200
140
110
90
68
55
40
30
20
13
8

## Slide 4
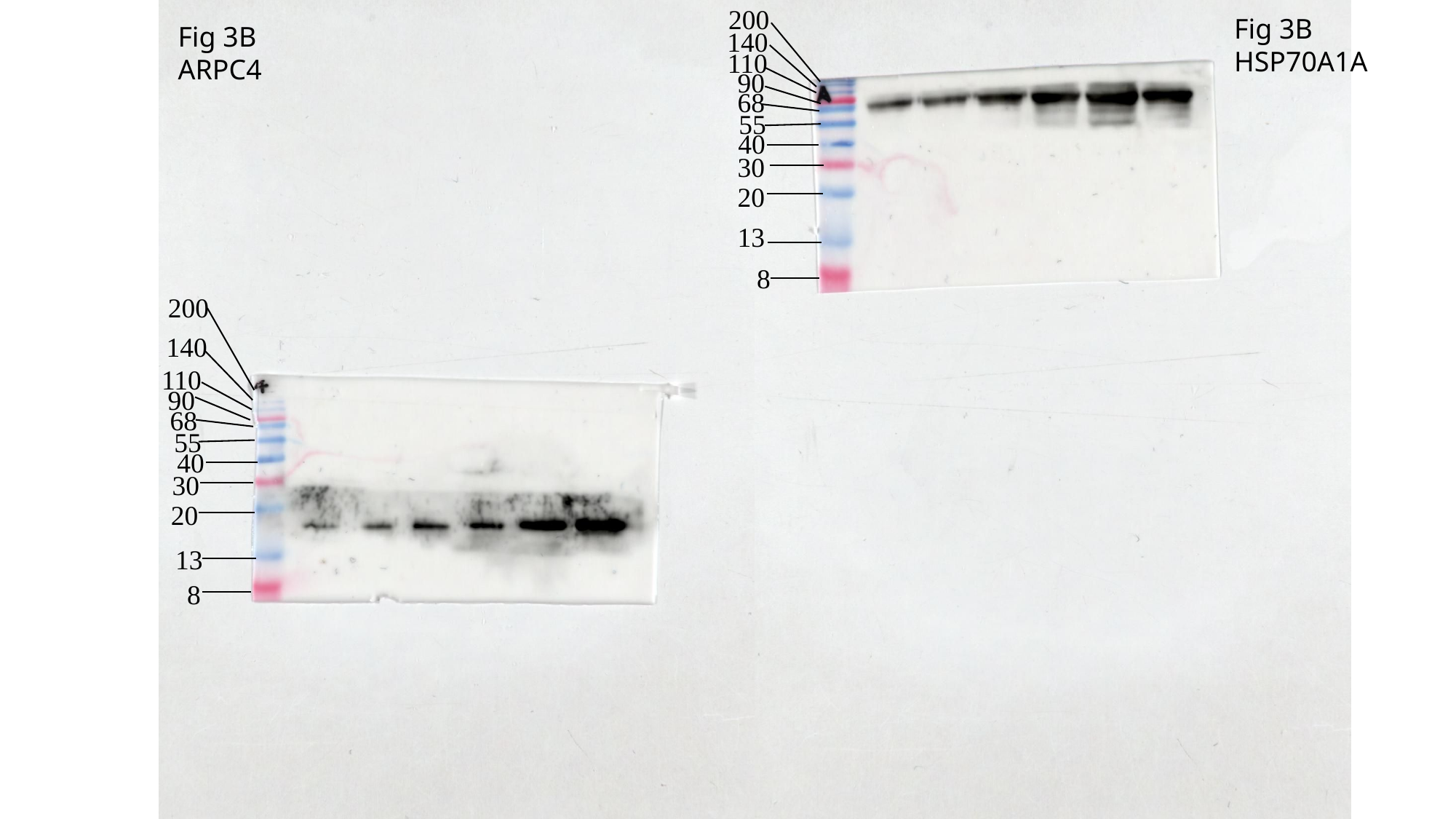

200
Fig 3B
HSP70A1A
Fig 3B
ARPC4
140
110
90
68
55
40
30
20
13
8
200
140
110
90
68
55
40
30
20
13
8

## Slide 5
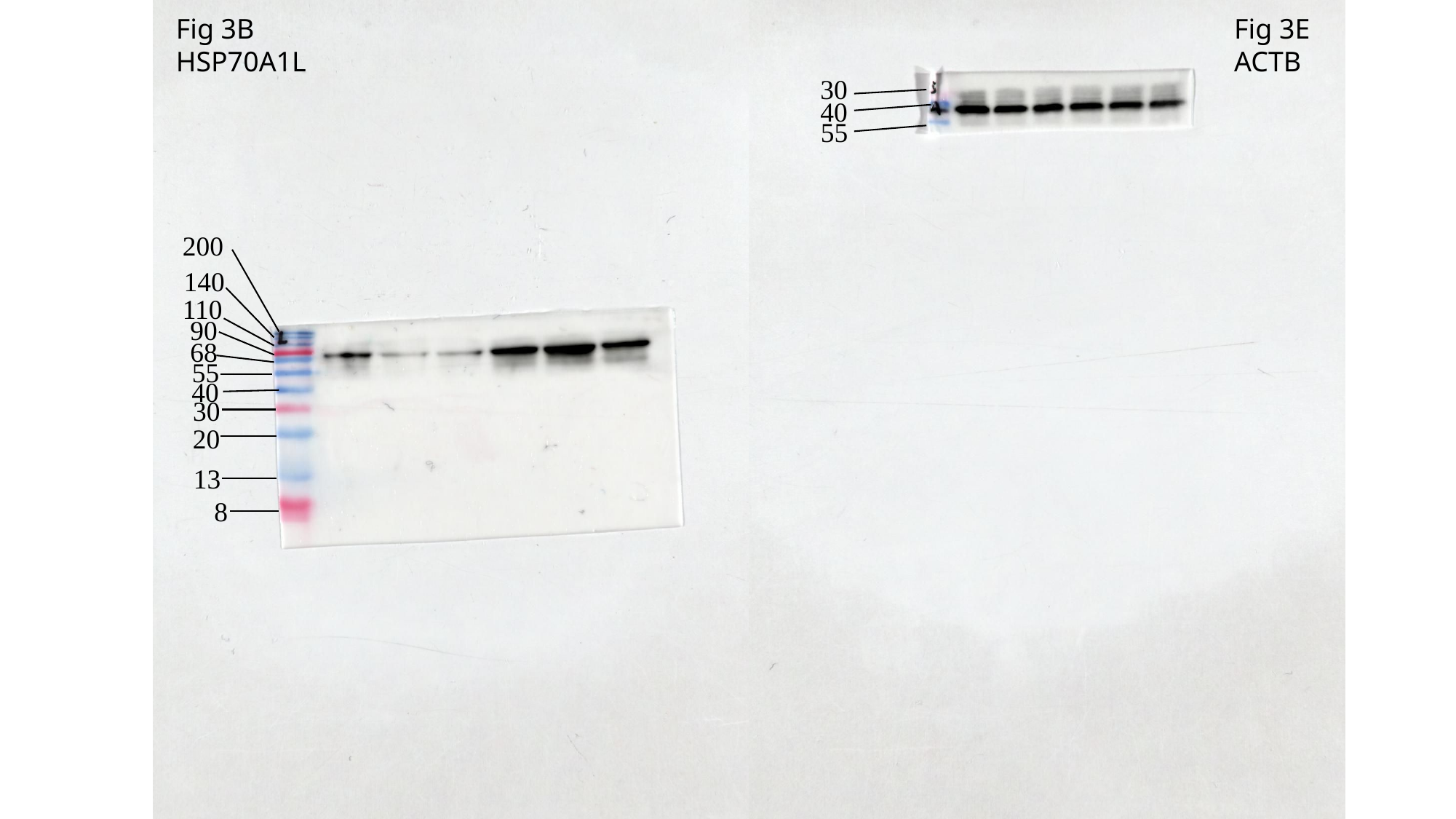

Fig 3B
HSP70A1L
Fig 3E
ACTB
30
40
55
200
140
110
90
68
55
40
30
20
13
8

## Slide 6
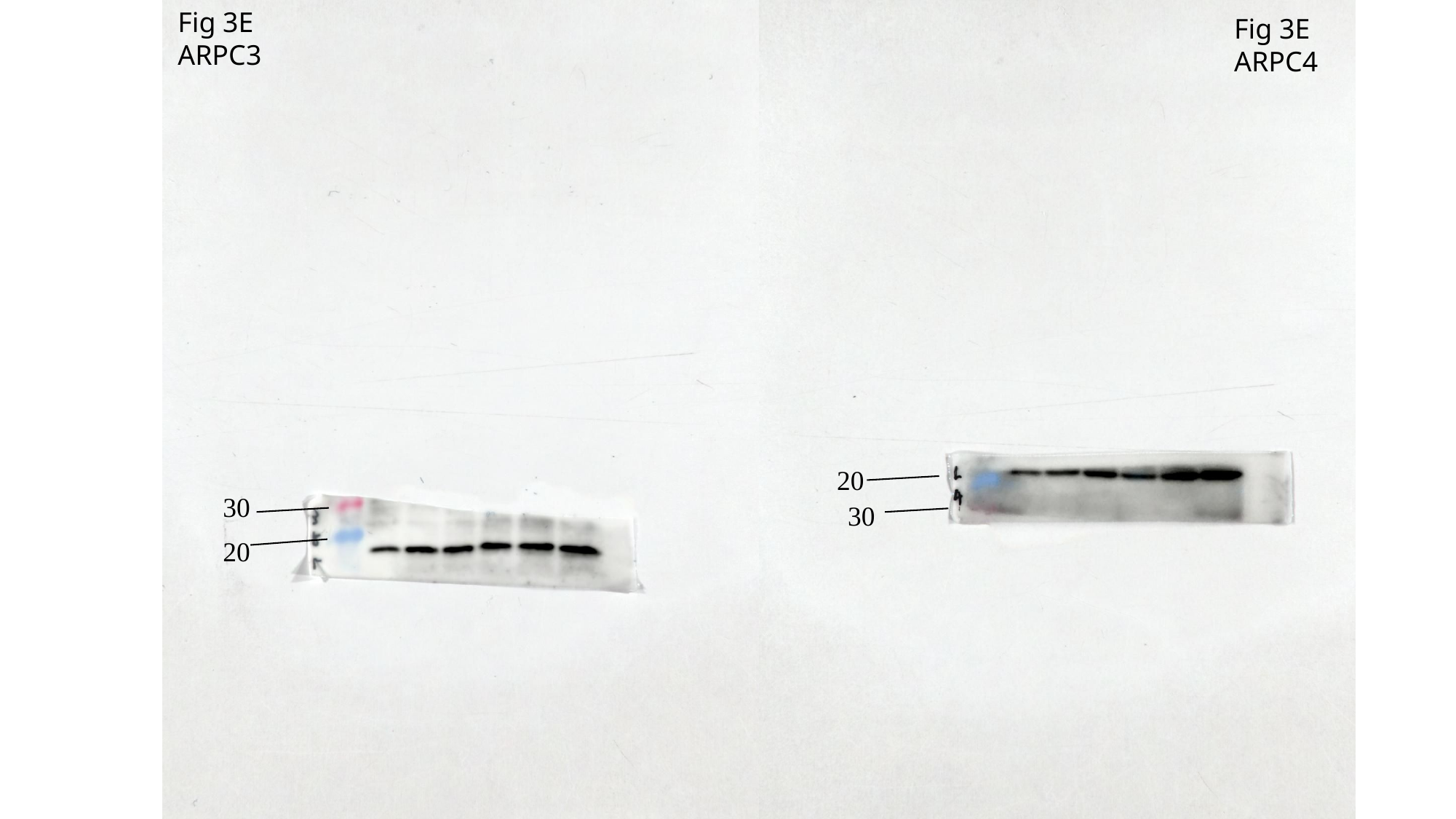

Fig 3E
ARPC3
Fig 3E
ARPC4
20
30
30
20

## Slide 7
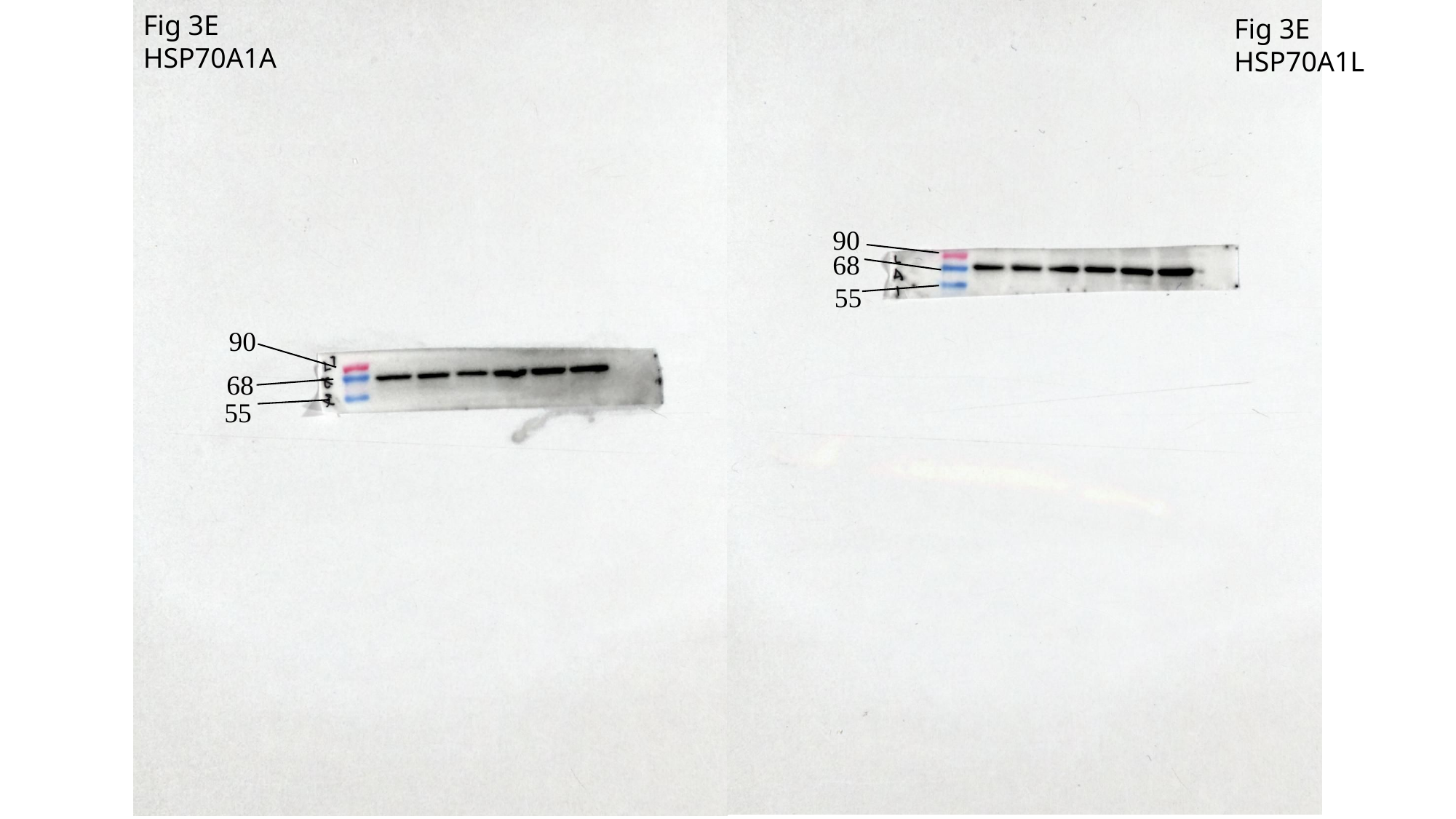

Fig 3E
HSP70A1A
Fig 3E
HSP70A1L
90
68
55
90
68
55

## Slide 8
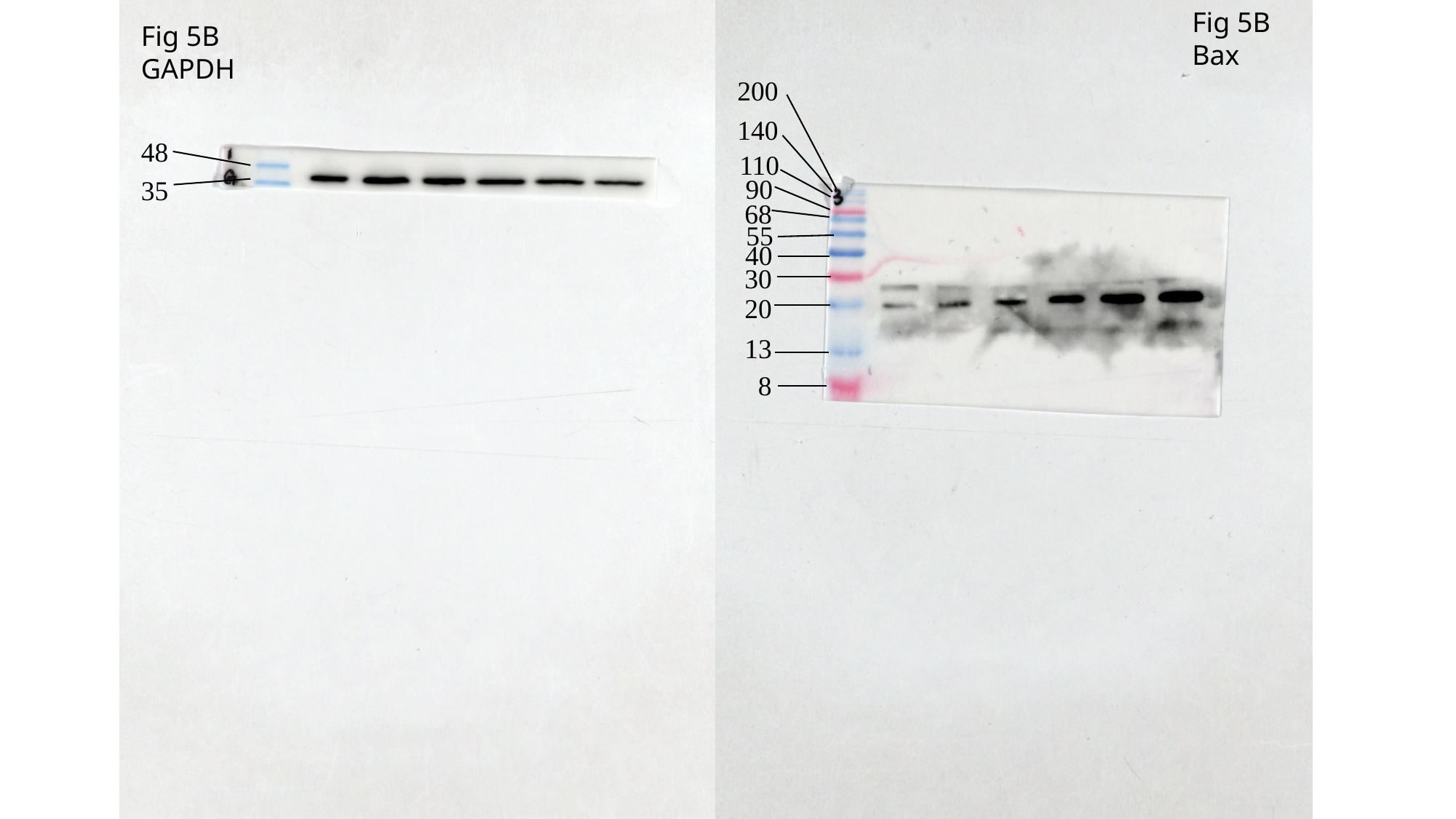

Fig 5B
Bax
Fig 5B
GAPDH
200
140
48
110
90
35
68
55
40
30
20
13
8

## Slide 9
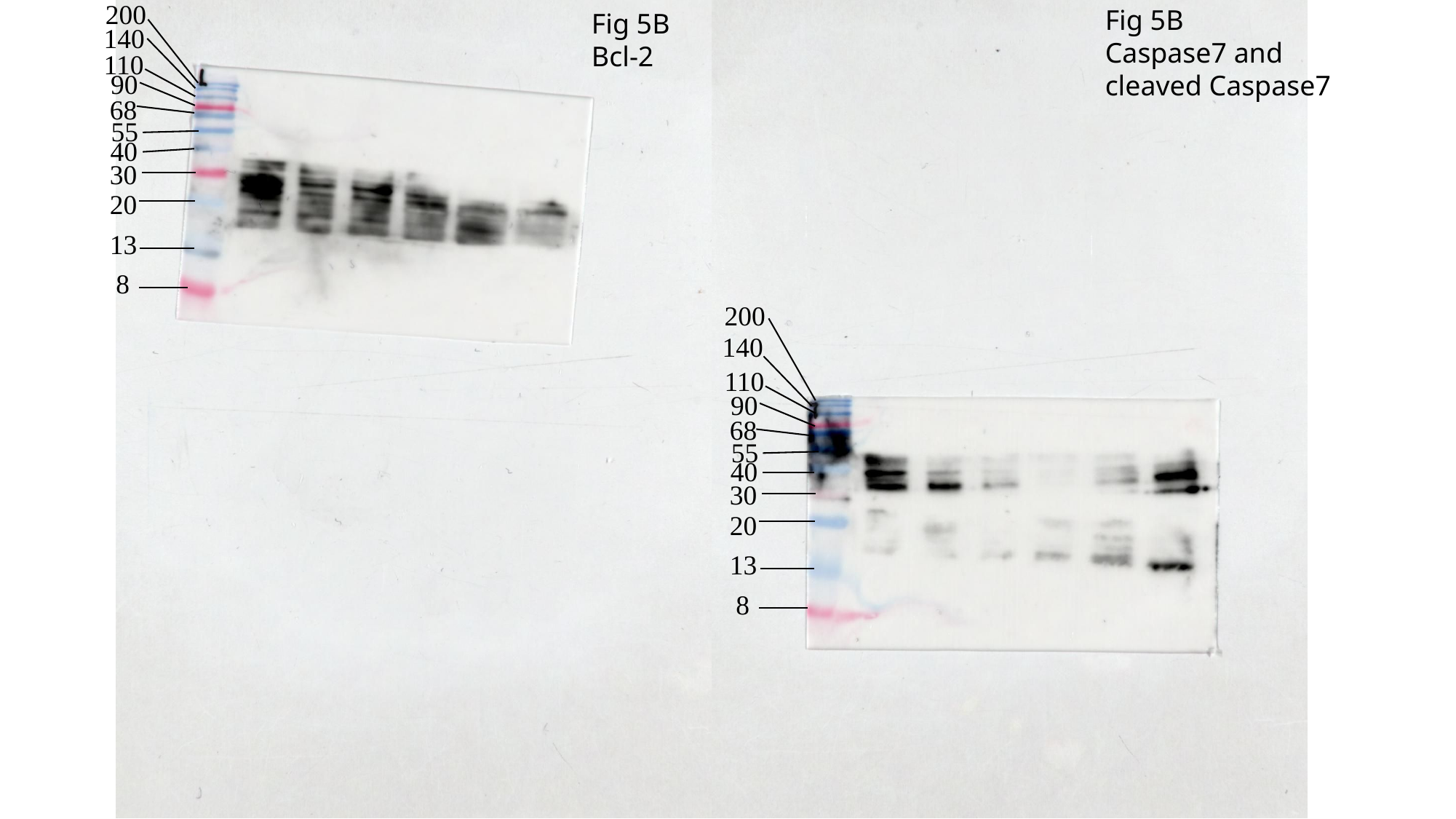

200
Fig 5B
Caspase7 and cleaved Caspase7
Fig 5B
Bcl-2
140
110
90
68
55
40
30
20
13
8
200
140
110
90
68
55
40
30
20
13
8

## Slide 10
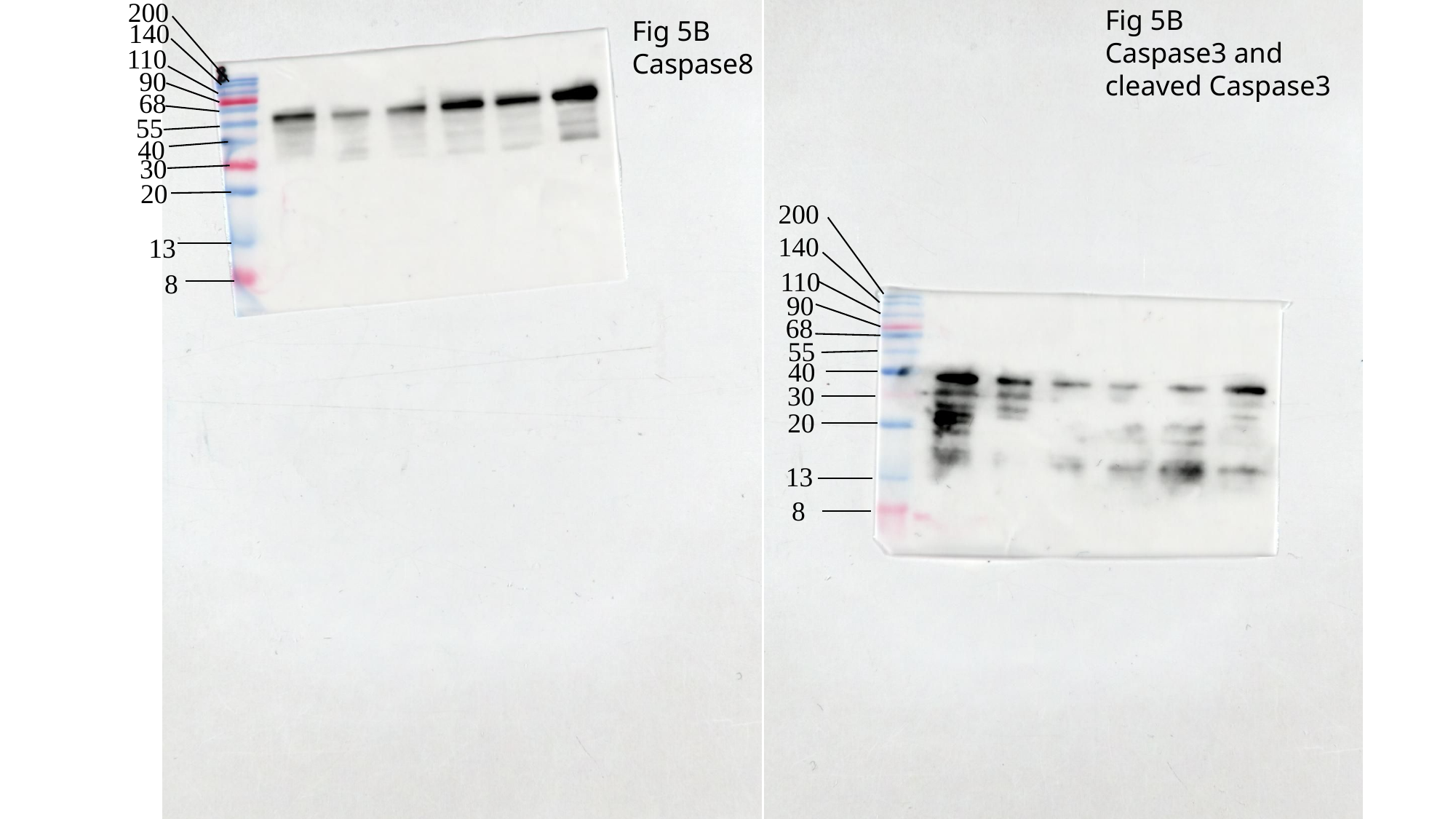

200
Fig 5B
Caspase3 and cleaved Caspase3
Fig 5B
Caspase8
140
110
90
68
55
40
30
20
200
140
13
110
8
90
68
55
40
30
20
13
8

## Slide 11
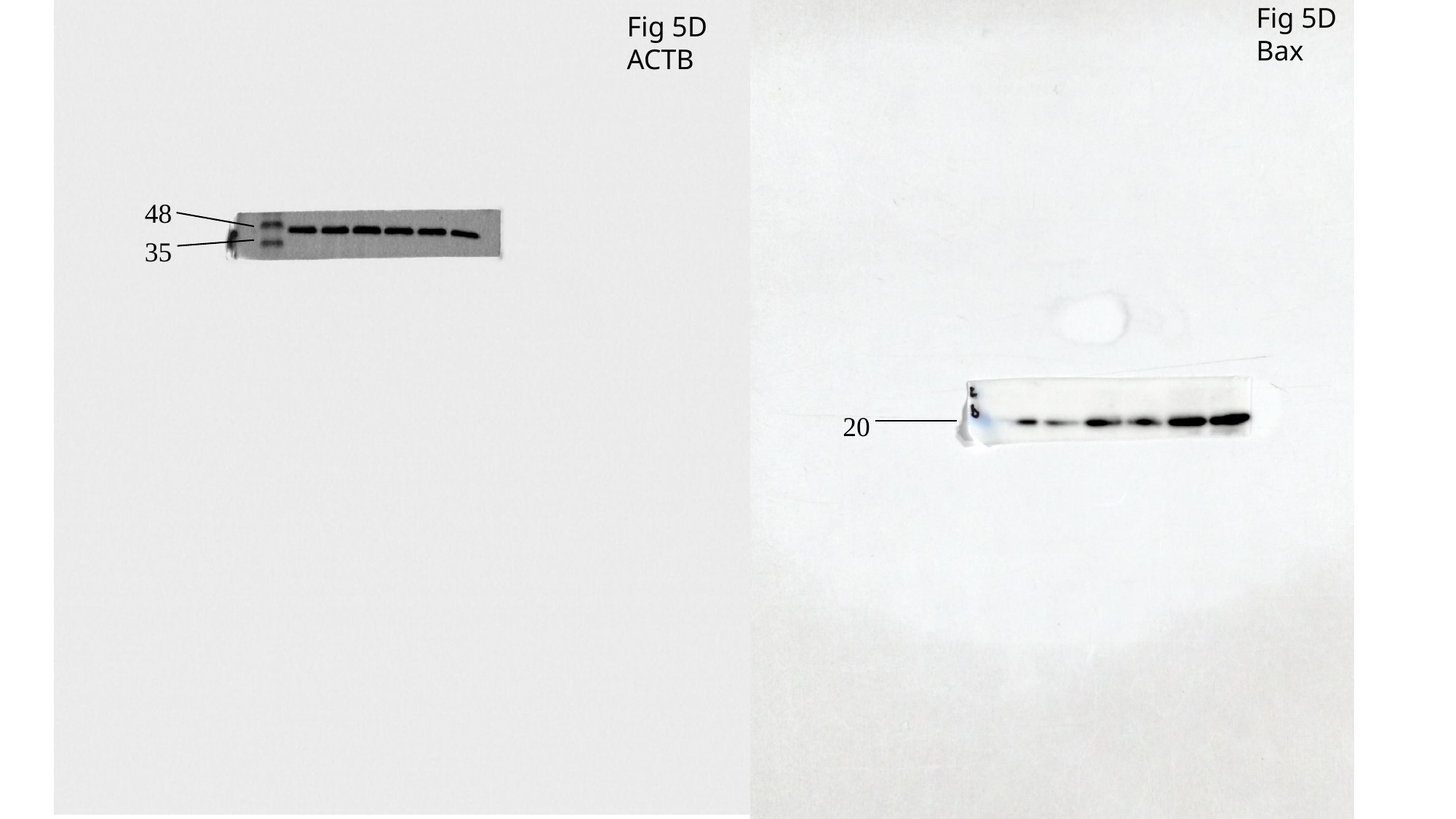

Fig 5D
Bax
Fig 5D
ACTB
48
35
20

## Slide 12
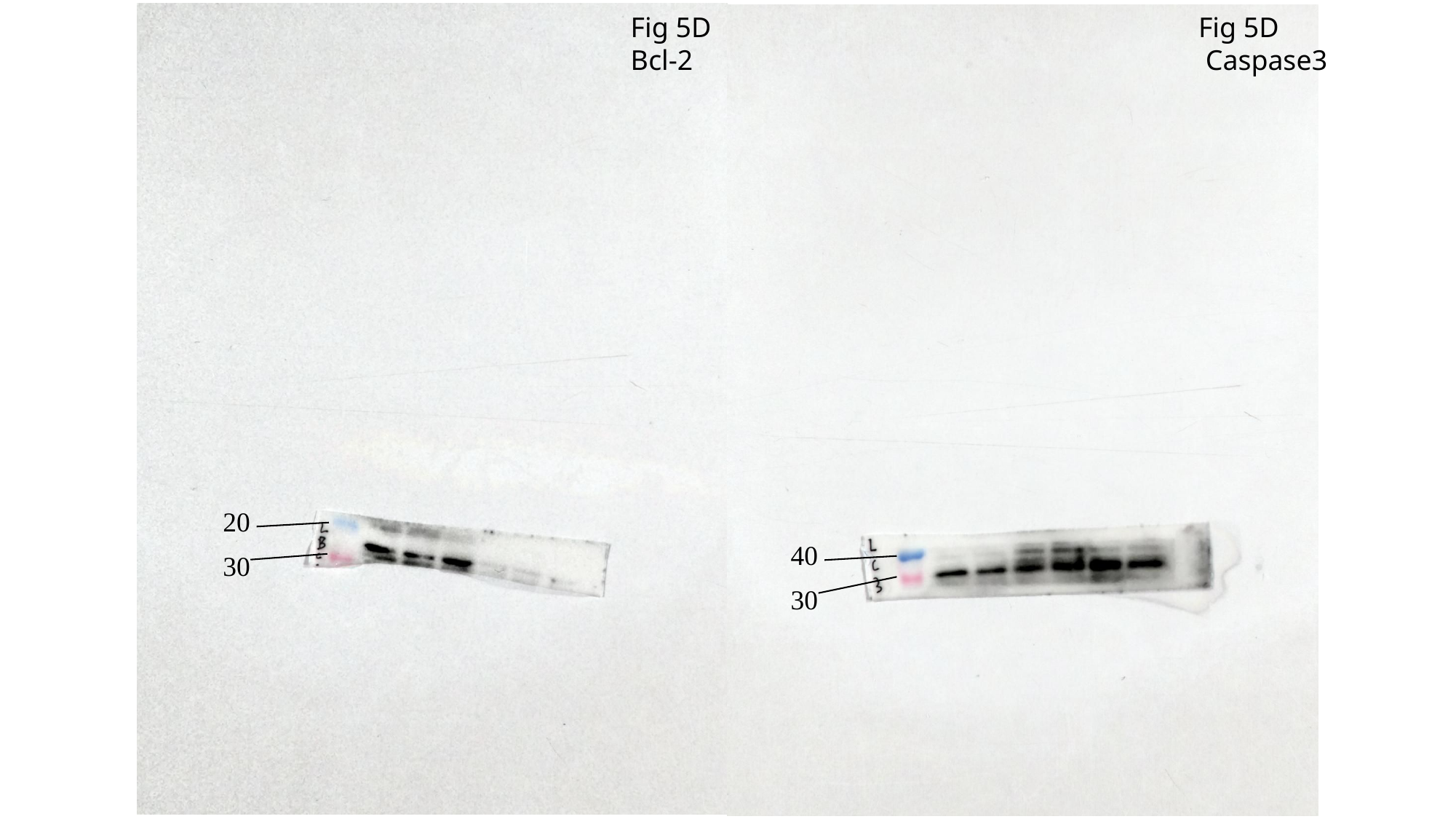

Fig 5D
Bcl-2
Fig 5D
 Caspase3
20
40
30
30

## Slide 13
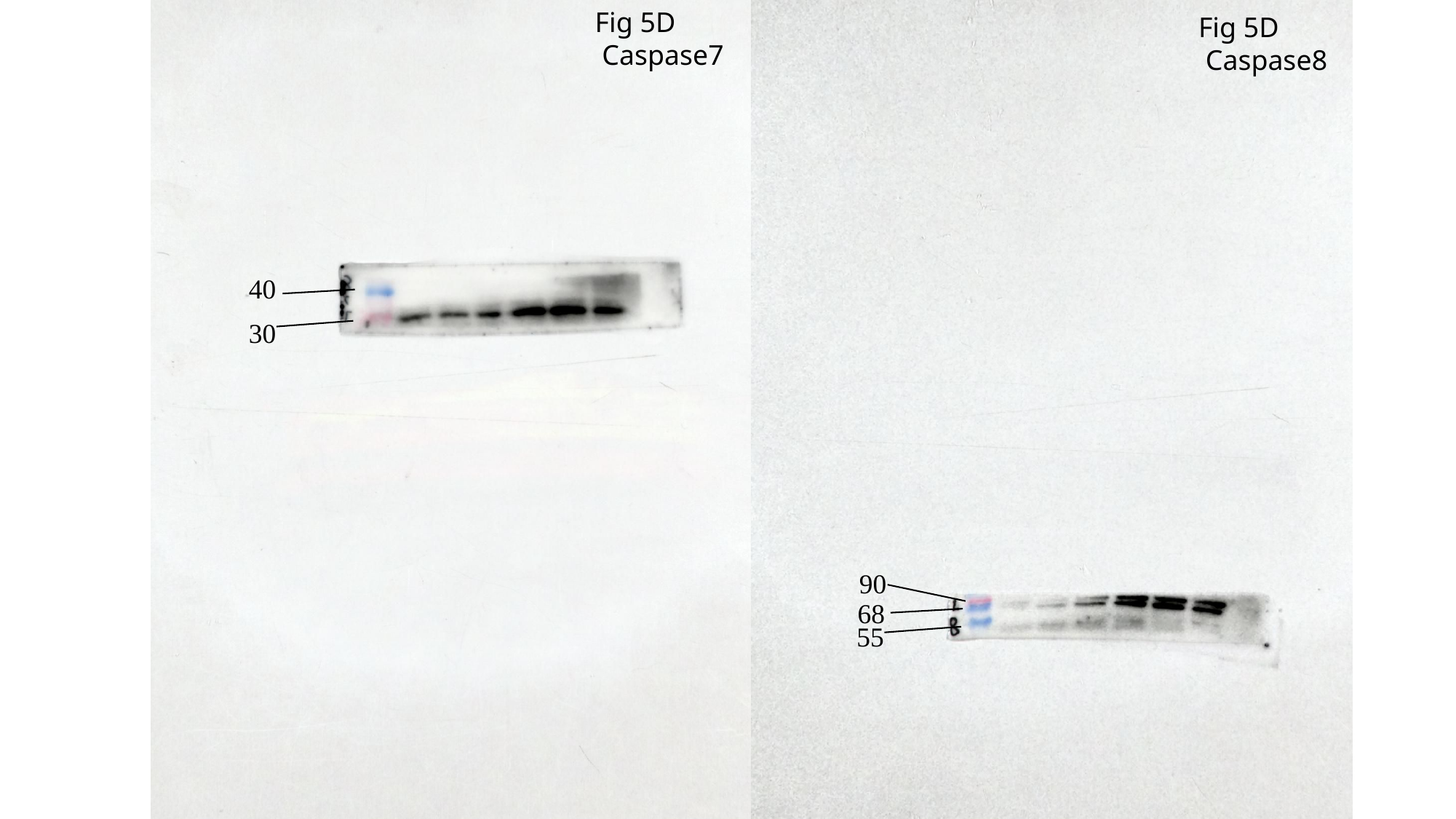

Fig 5D
 Caspase7
Fig 5D
 Caspase8
40
30
90
68
55
