# Supplementary figures and images for: Activation of ARP2/3 and HSP70 Expression by Lipoteichoic Acid: Potential Bidirectional Regulation of Apoptosis in a Mastitis Inflammation Model
Source: Biomolecules. 2024 Jul 25;14(8):901. doi: 10.3390/biom14080901 (PMC11352453; doi:10.3390/biom14080901)

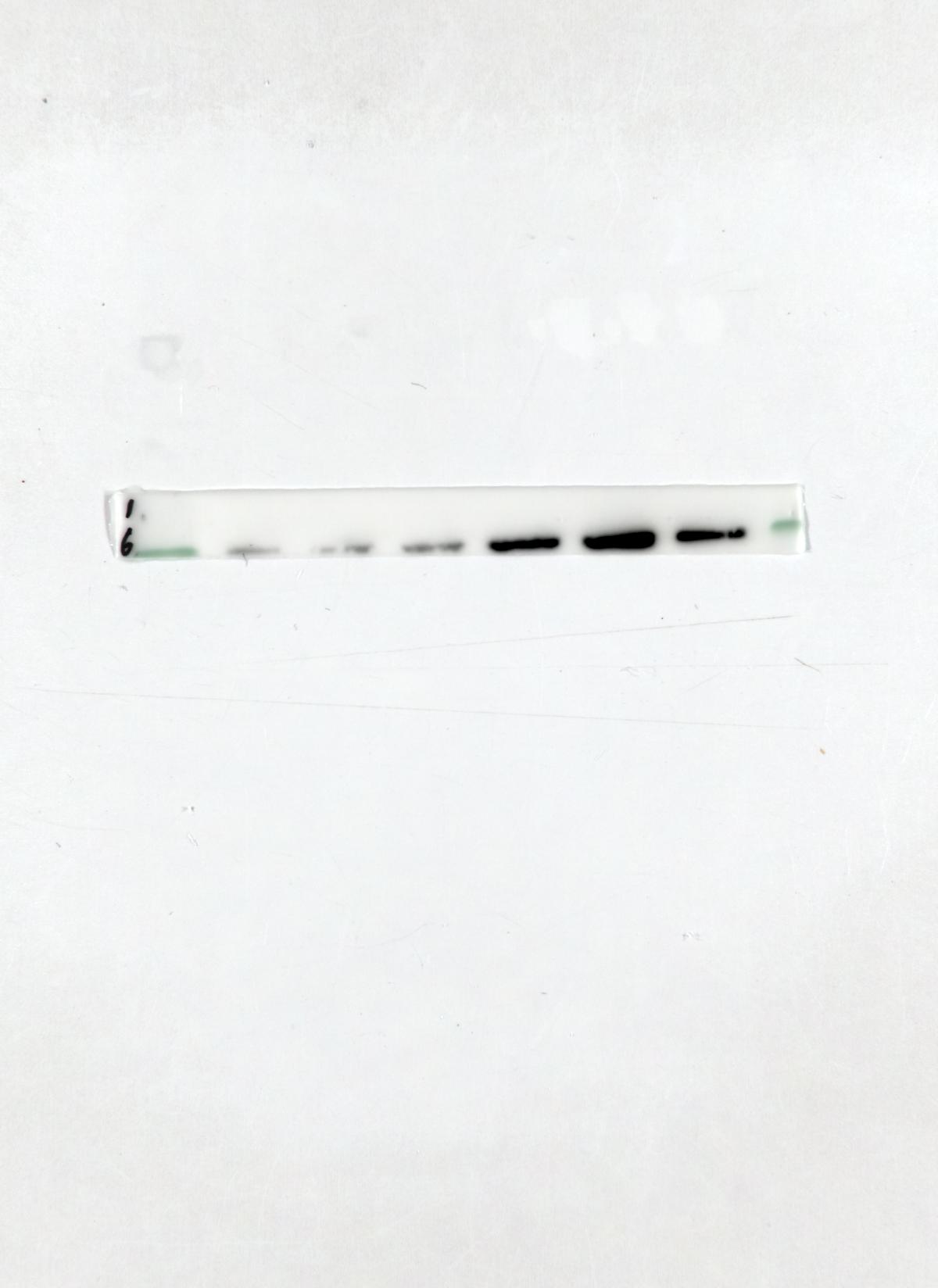

Supplement: Supplementary file 1 [file biomolecules-14-00901-s001.zip › Fig.1 E IL-6.jpg]

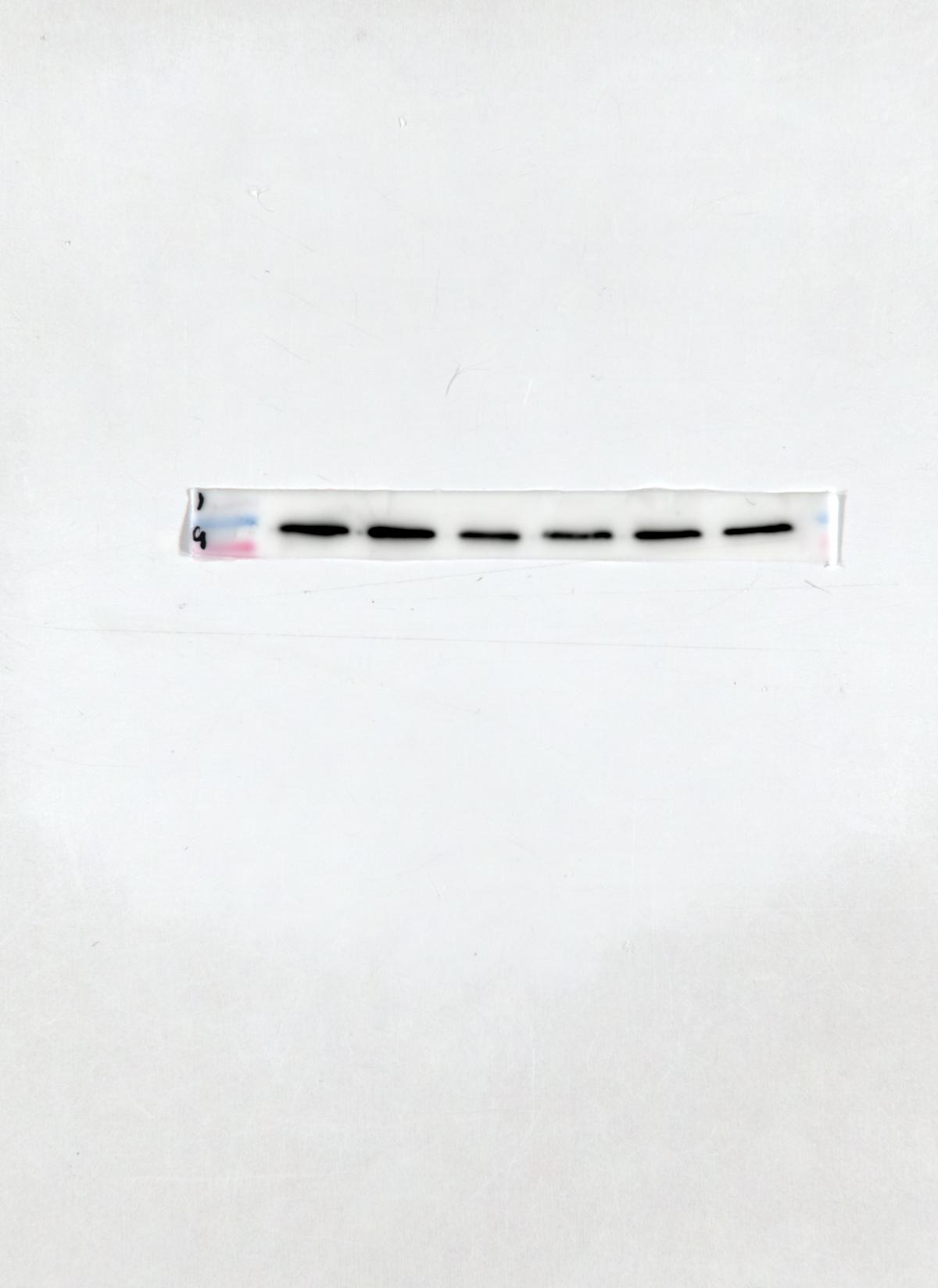

Supplement: Supplementary file 1 [file biomolecules-14-00901-s001.zip › Fig.1E GAPDH.jpg]

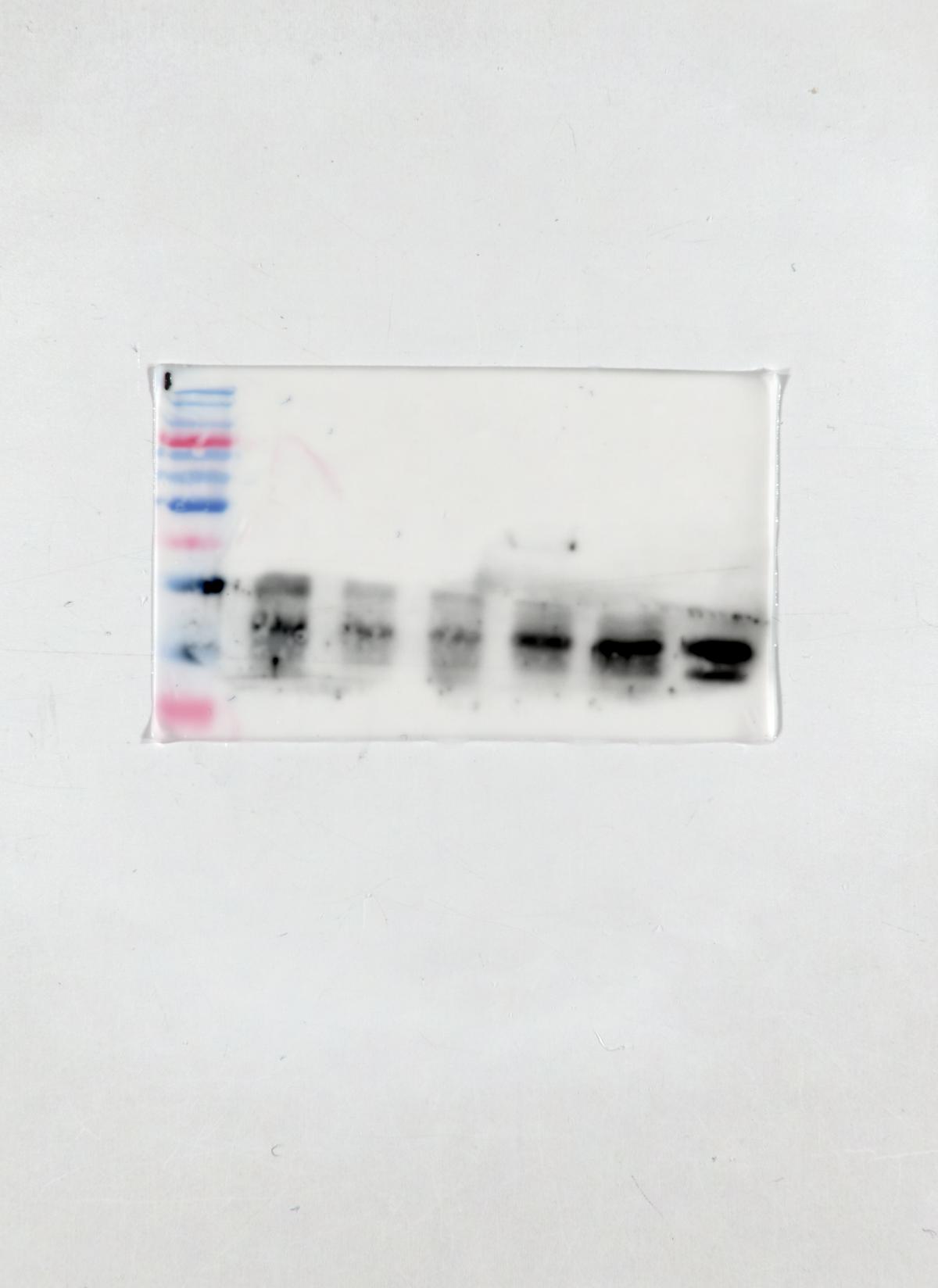

Supplement: Supplementary file 1 [file biomolecules-14-00901-s001.zip › Fig.1E IL-1beta.jpg]

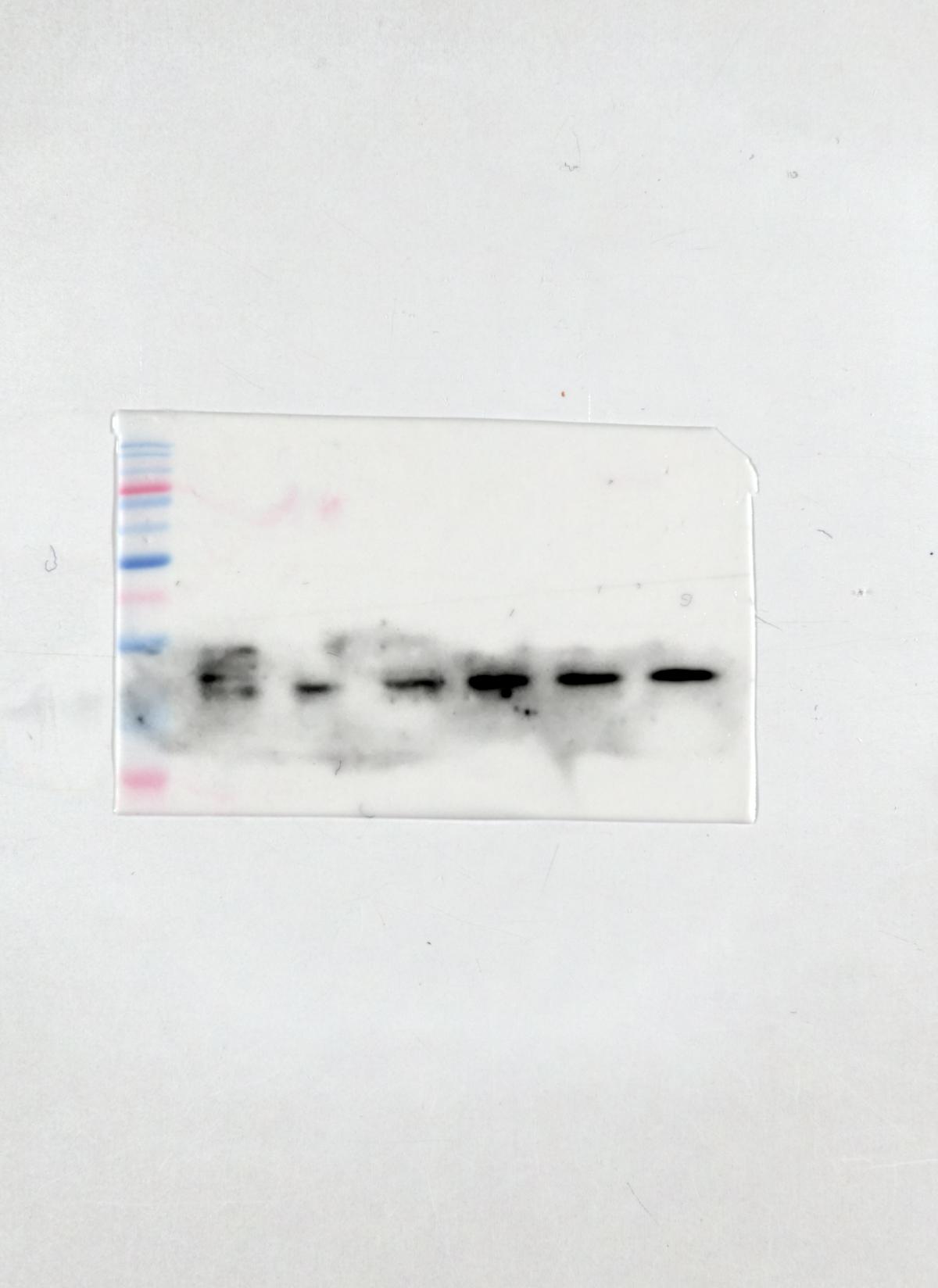

Supplement: Supplementary file 1 [file biomolecules-14-00901-s001.zip › Fig.1E TNF-alpha.jpg]

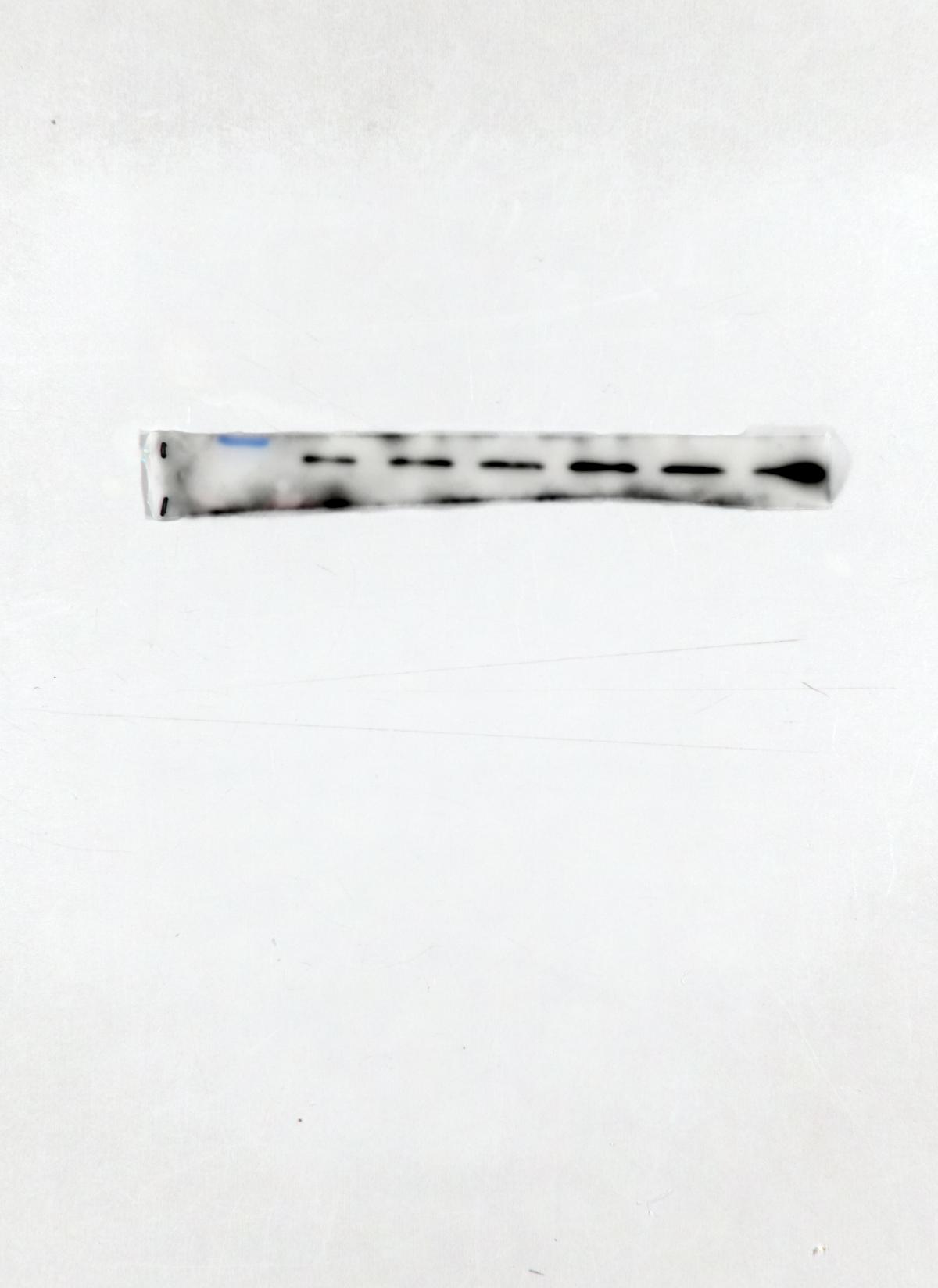

Supplement: Supplementary file 1 [file biomolecules-14-00901-s001.zip › Fig.3B ARPC3.jpg]

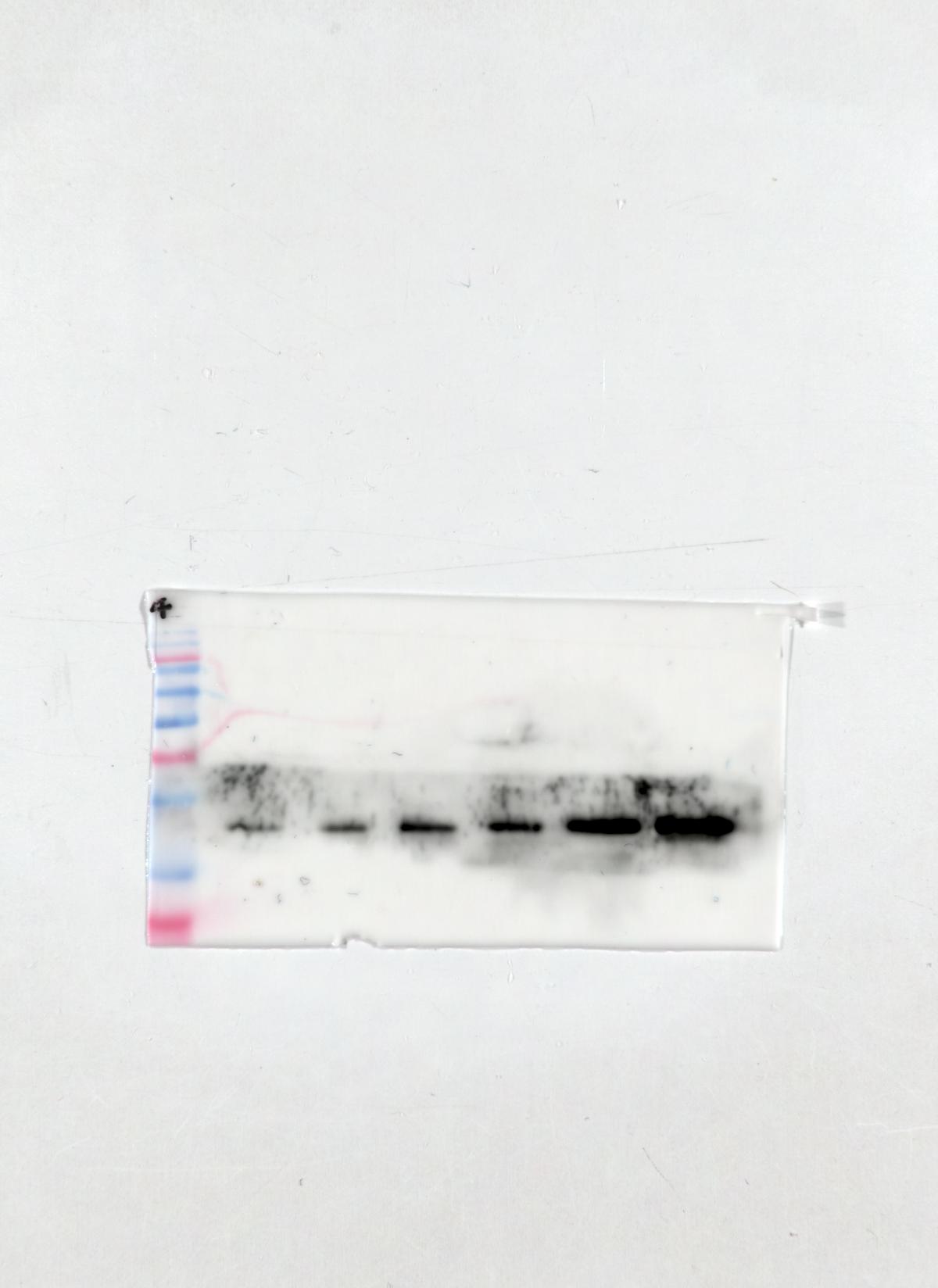

Supplement: Supplementary file 1 [file biomolecules-14-00901-s001.zip › Fig.3B ARPC4.jpg]

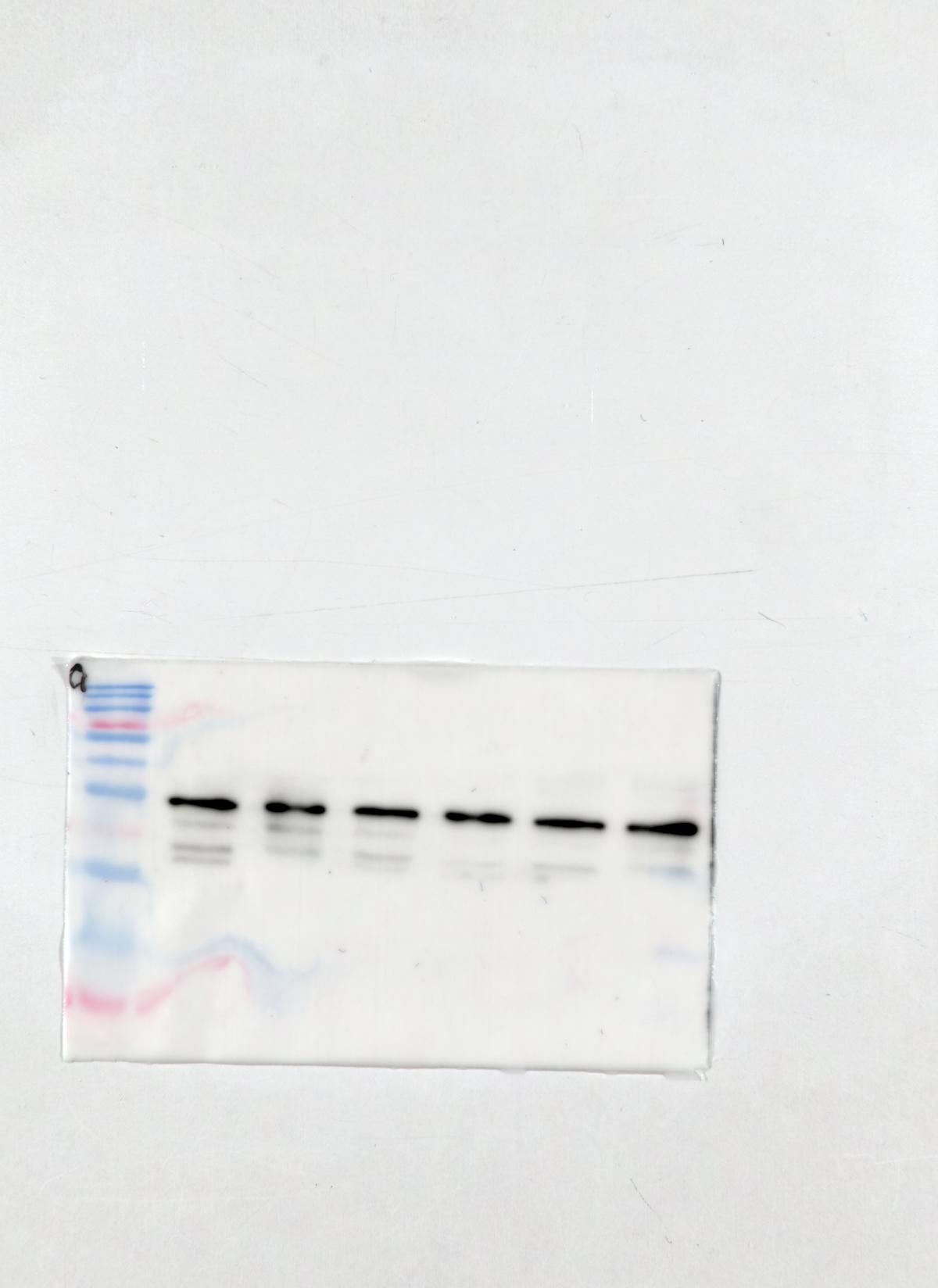

Supplement: Supplementary file 1 [file biomolecules-14-00901-s001.zip › Fig.3B GAPDH.jpg]

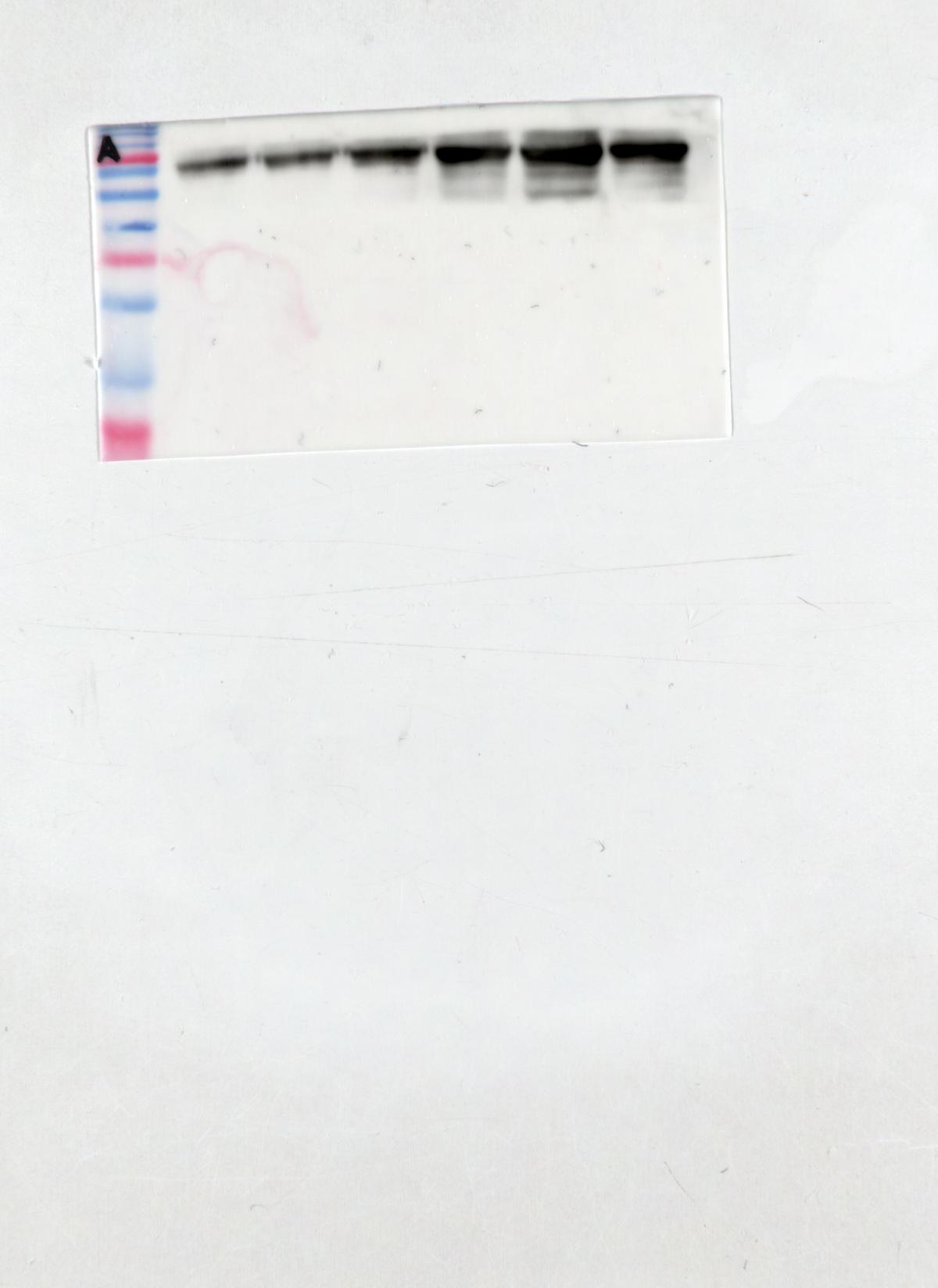

Supplement: Supplementary file 1 [file biomolecules-14-00901-s001.zip › Fig.3B HSP70A1A.jpg]

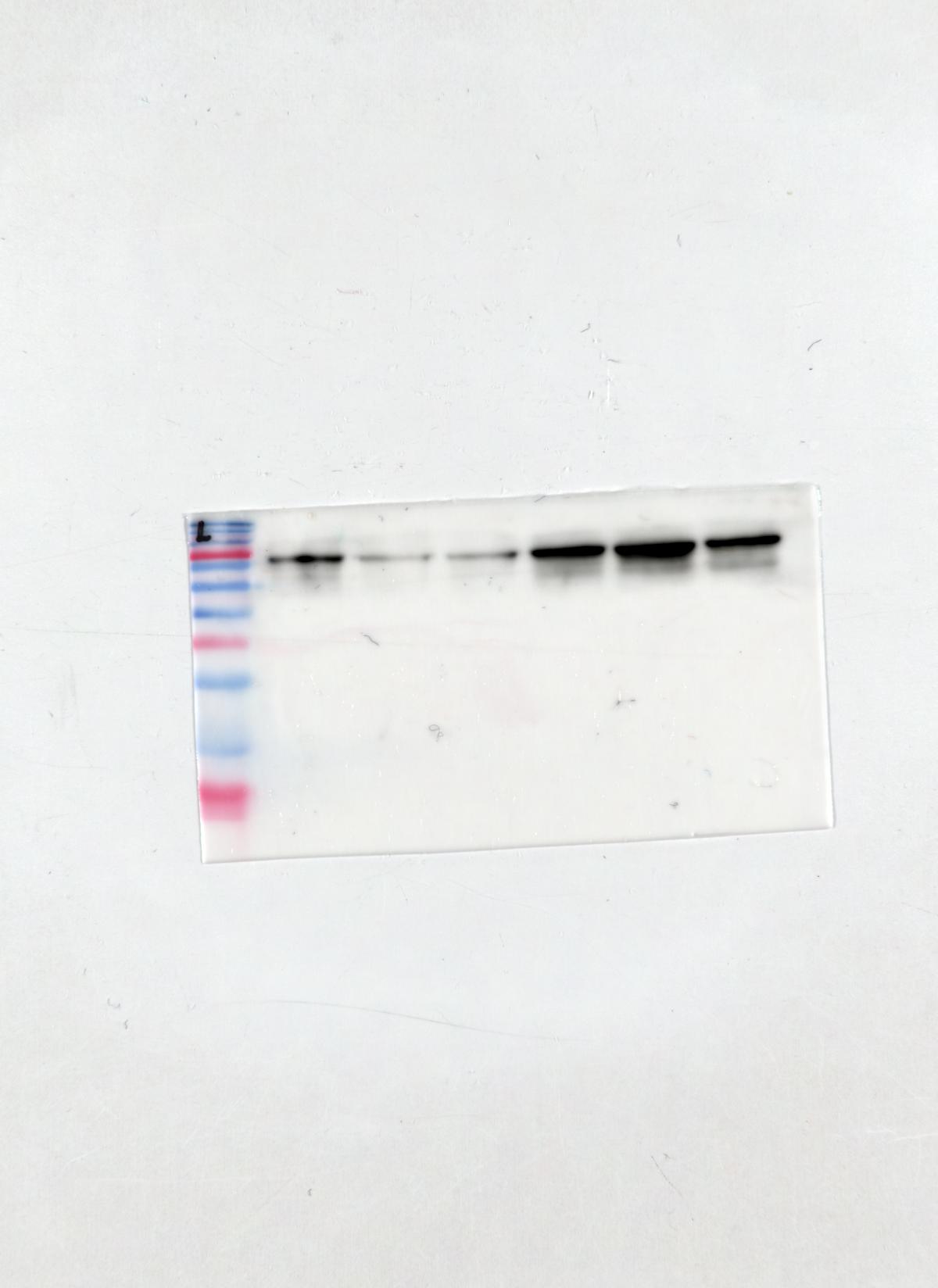

Supplement: Supplementary file 1 [file biomolecules-14-00901-s001.zip › Fig.3B HSP70A1L.jpg]

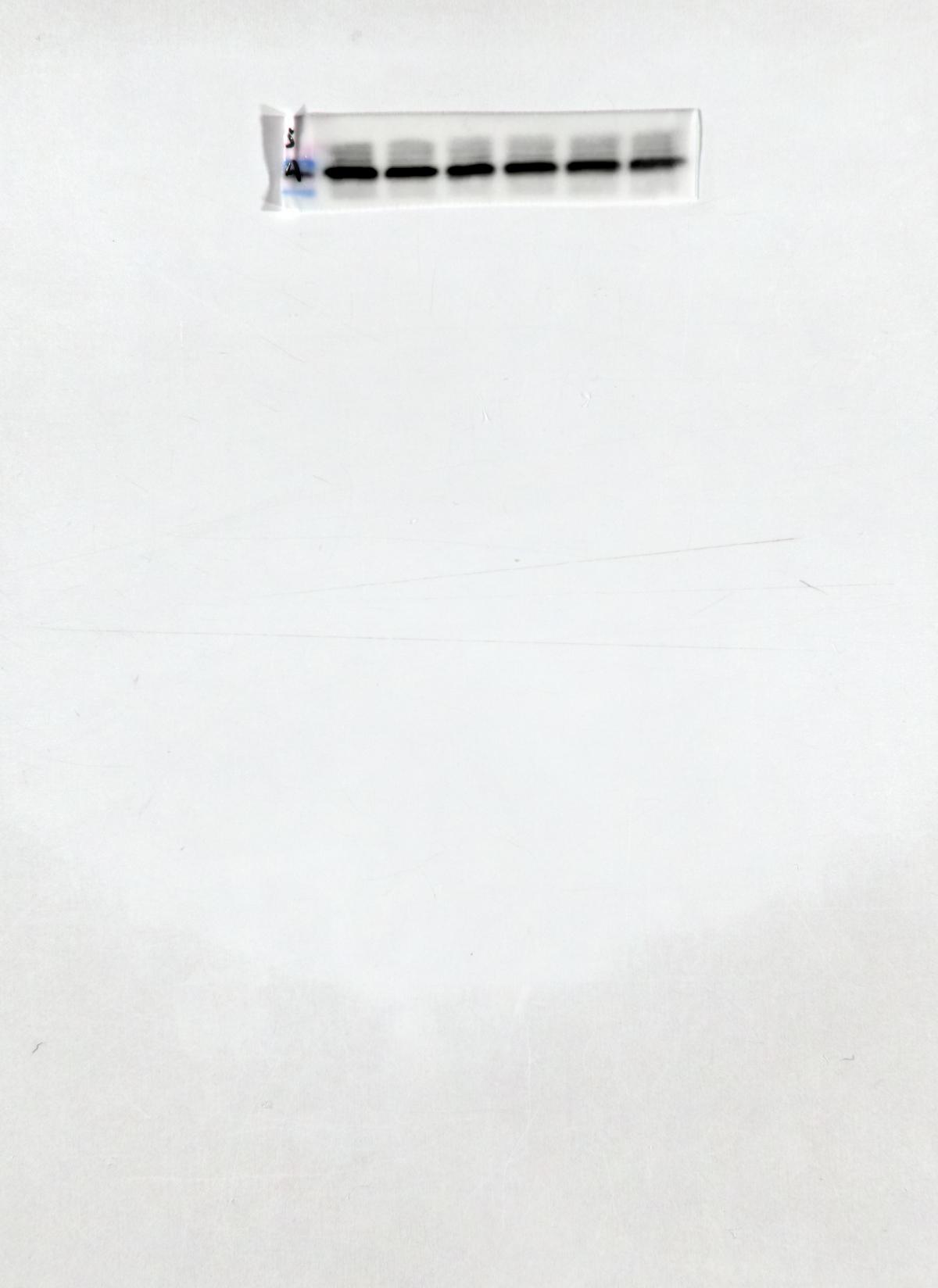

Supplement: Supplementary file 1 [file biomolecules-14-00901-s001.zip › Fig.3E ACTB.jpg]

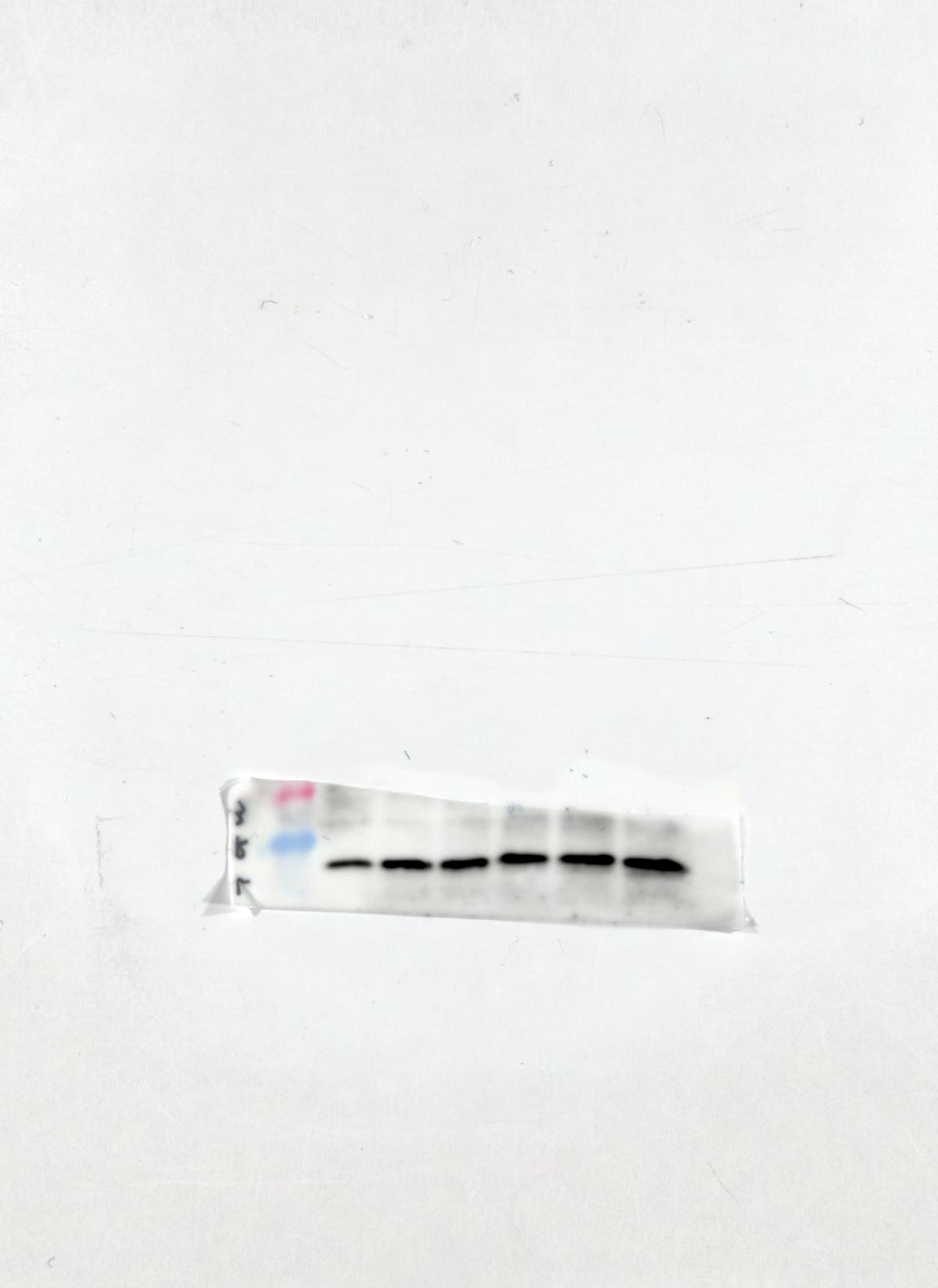

Supplement: Supplementary file 1 [file biomolecules-14-00901-s001.zip › Fig.3E ARPC3.jpg]

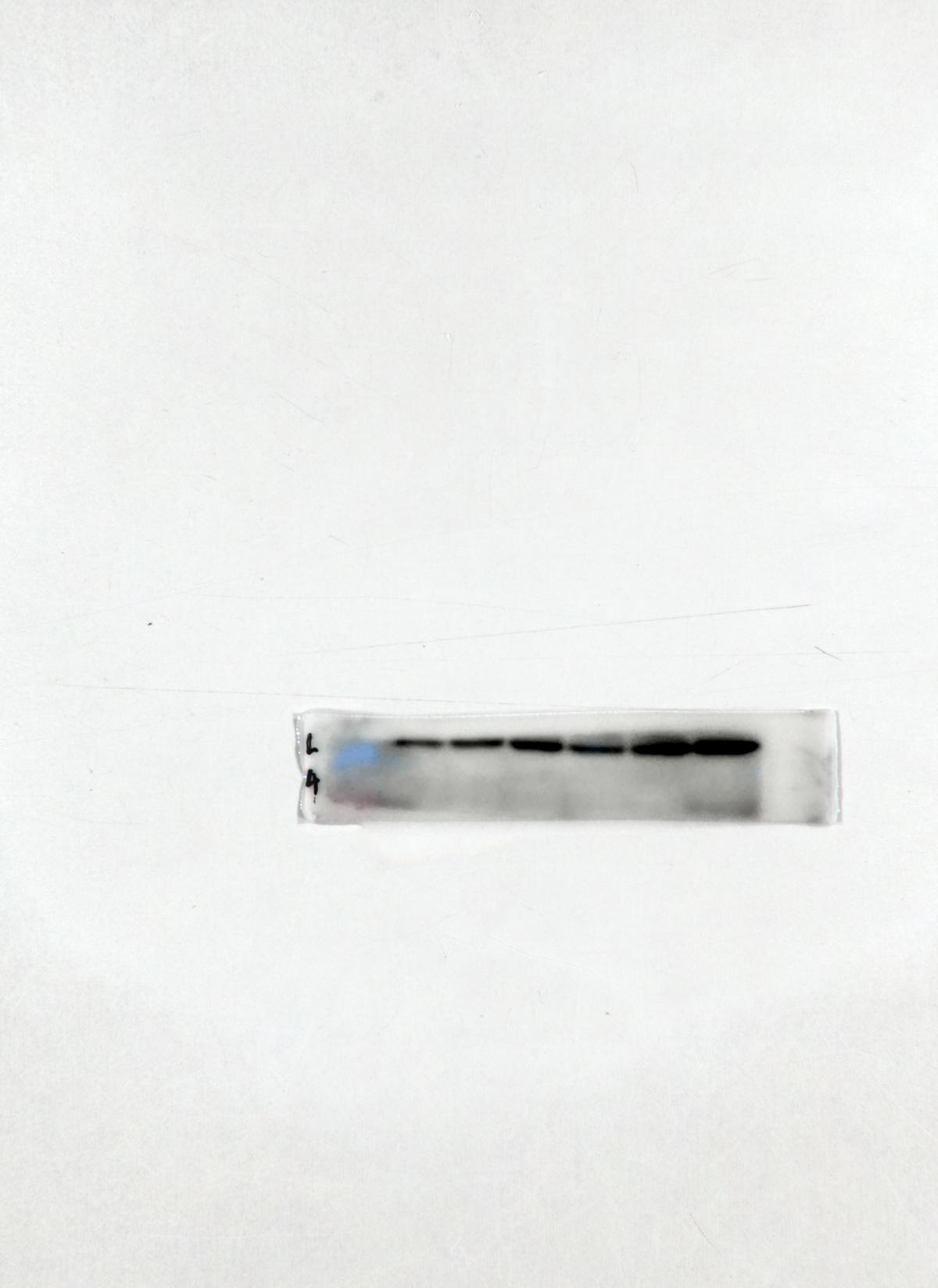

Supplement: Supplementary file 1 [file biomolecules-14-00901-s001.zip › Fig.3E ARPC4.jpg]

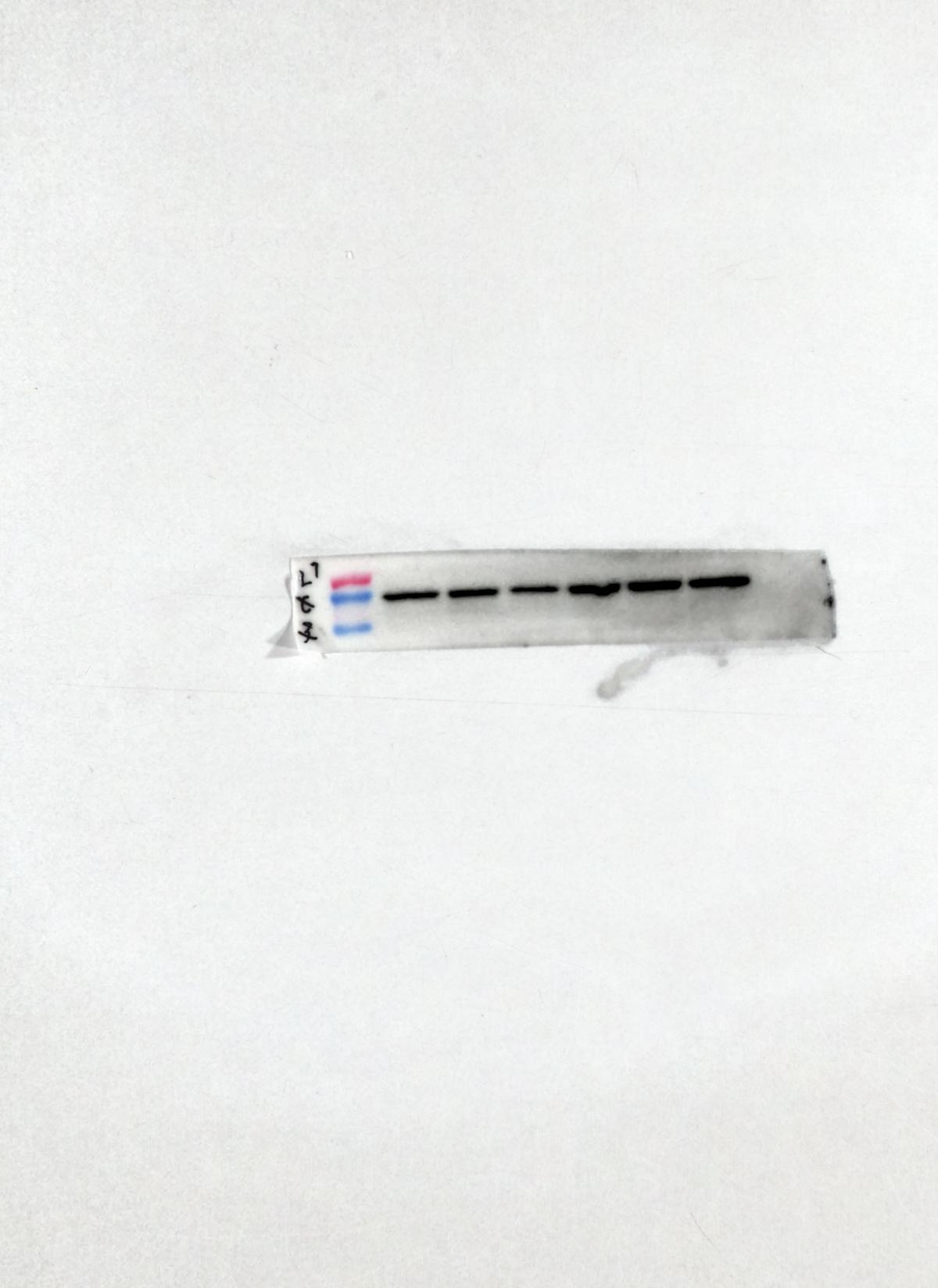

Supplement: Supplementary file 1 [file biomolecules-14-00901-s001.zip › Fig.3E HSP70A1A.jpg]

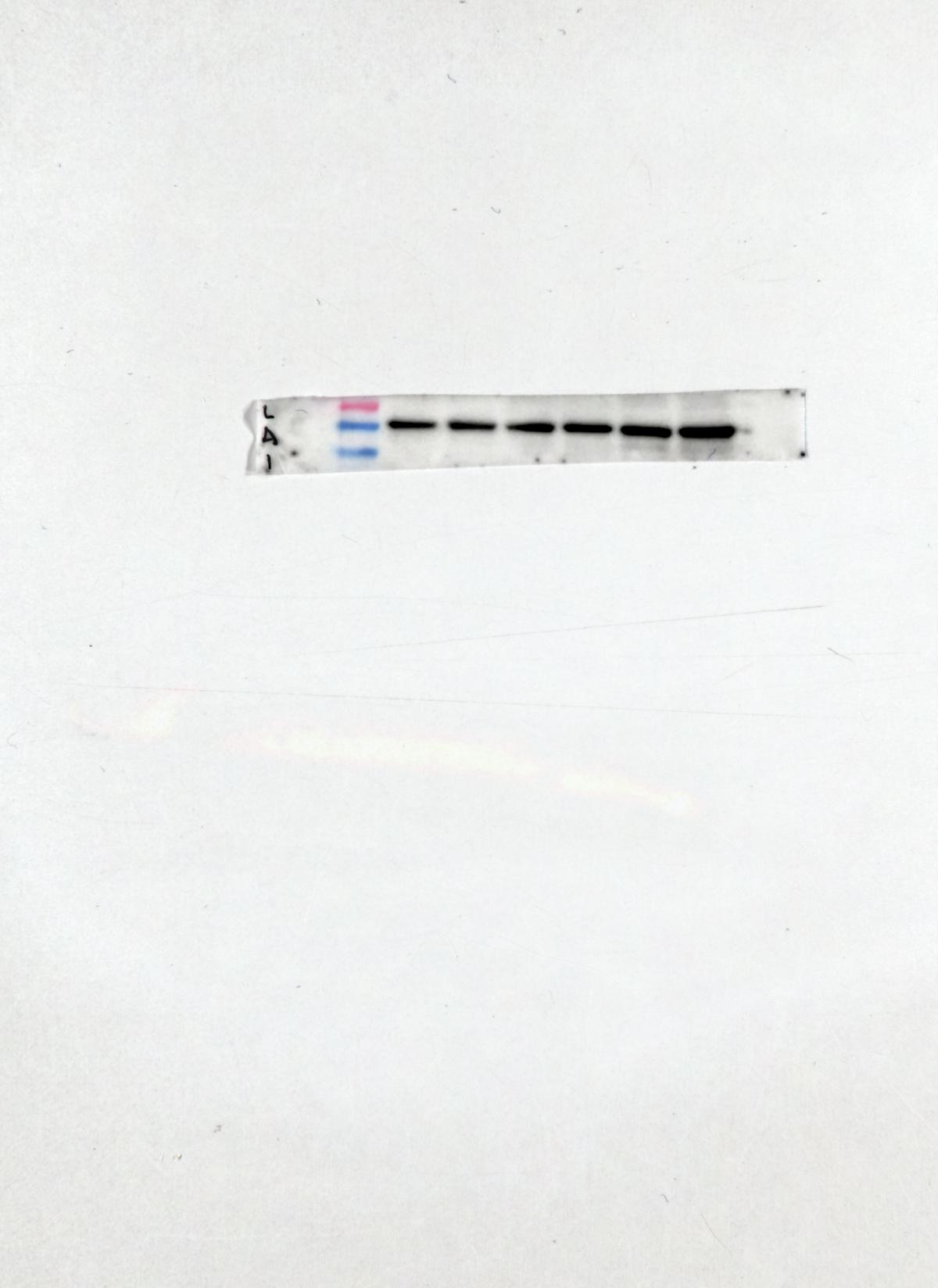

Supplement: Supplementary file 1 [file biomolecules-14-00901-s001.zip › Fig.3E HSP70A1L.jpg]

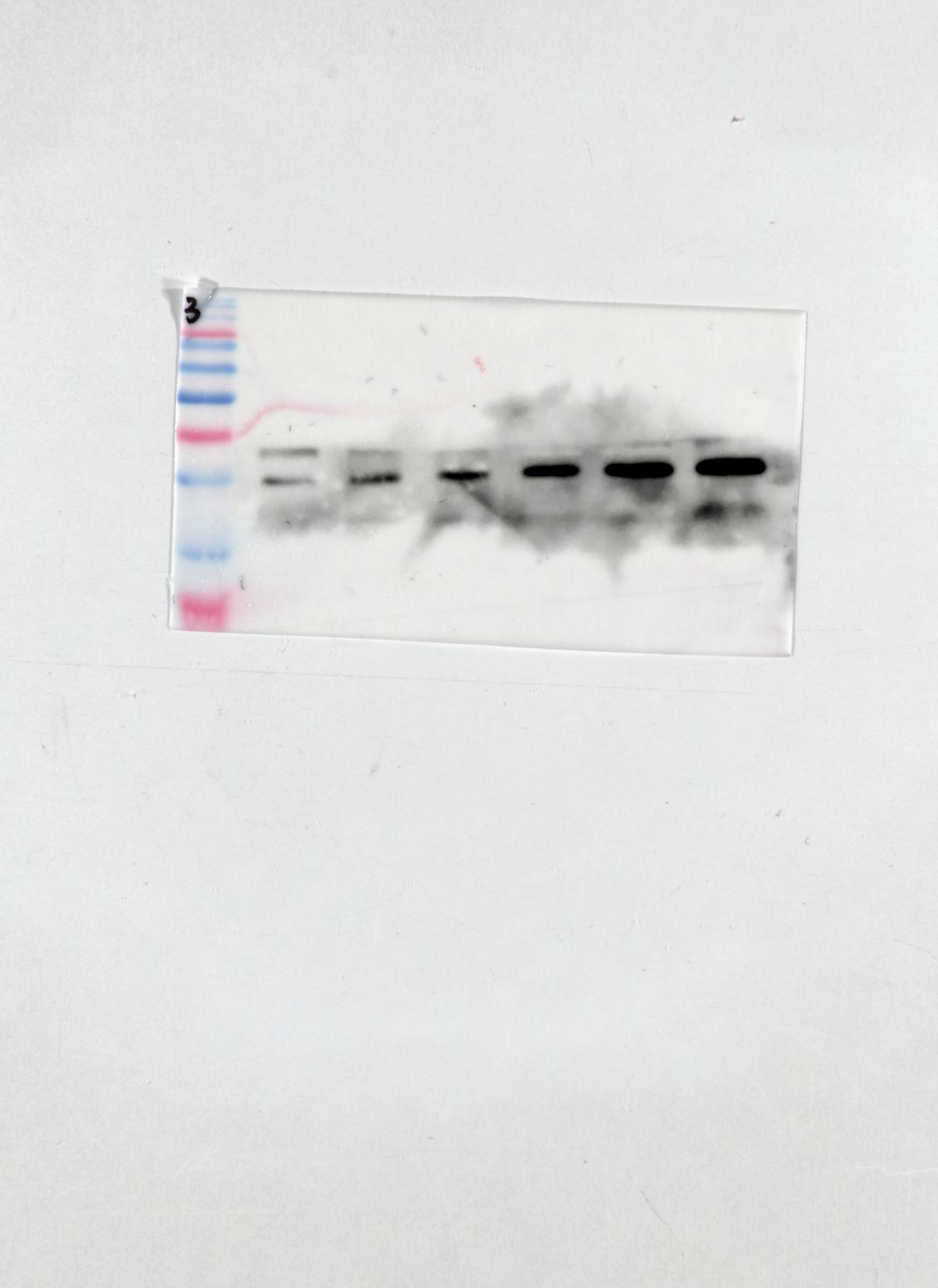

Supplement: Supplementary file 1 [file biomolecules-14-00901-s001.zip › Fig.5B Bax.jpg]

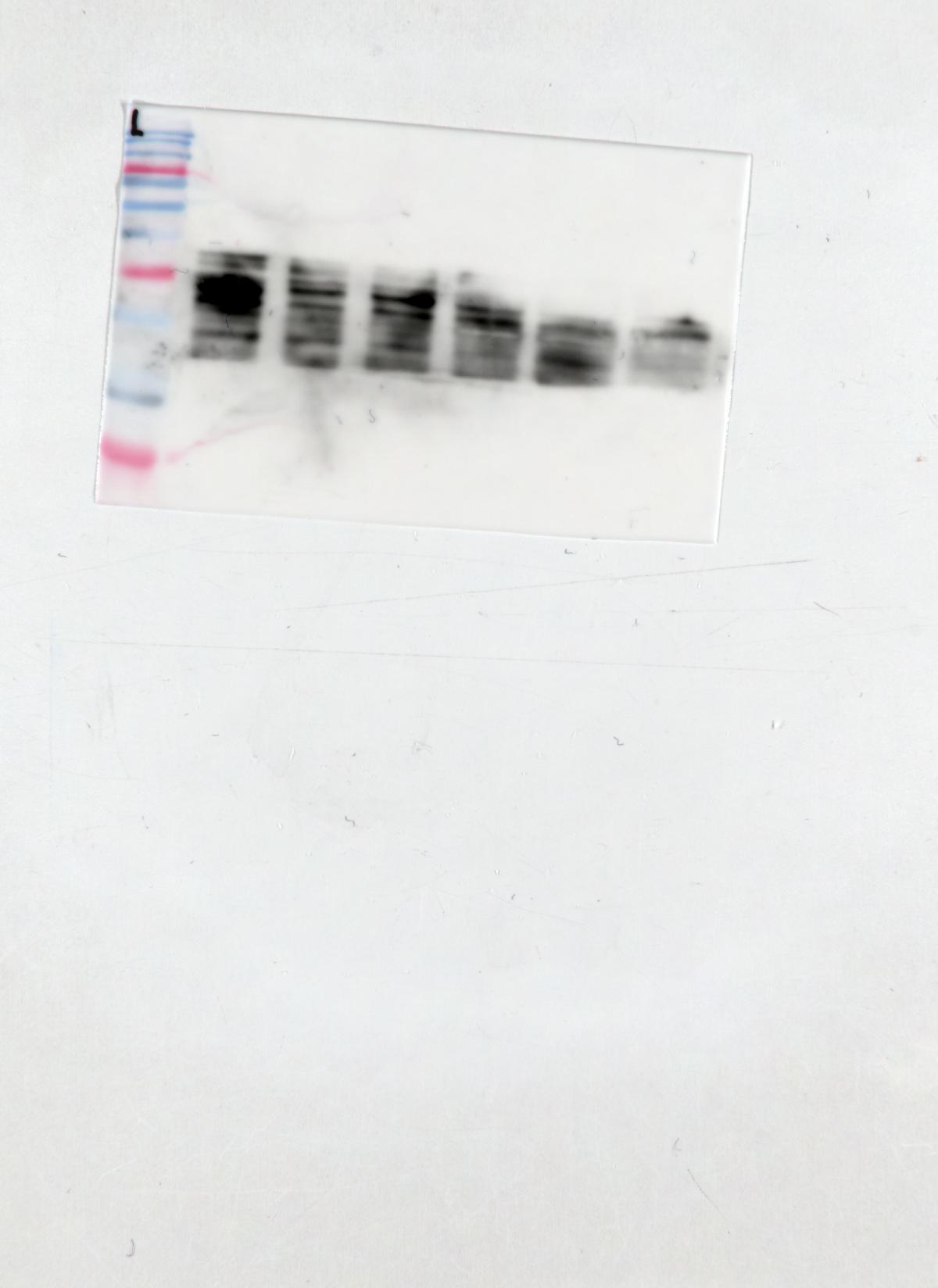

Supplement: Supplementary file 1 [file biomolecules-14-00901-s001.zip › Fig.5B Bcl-2.jpg]

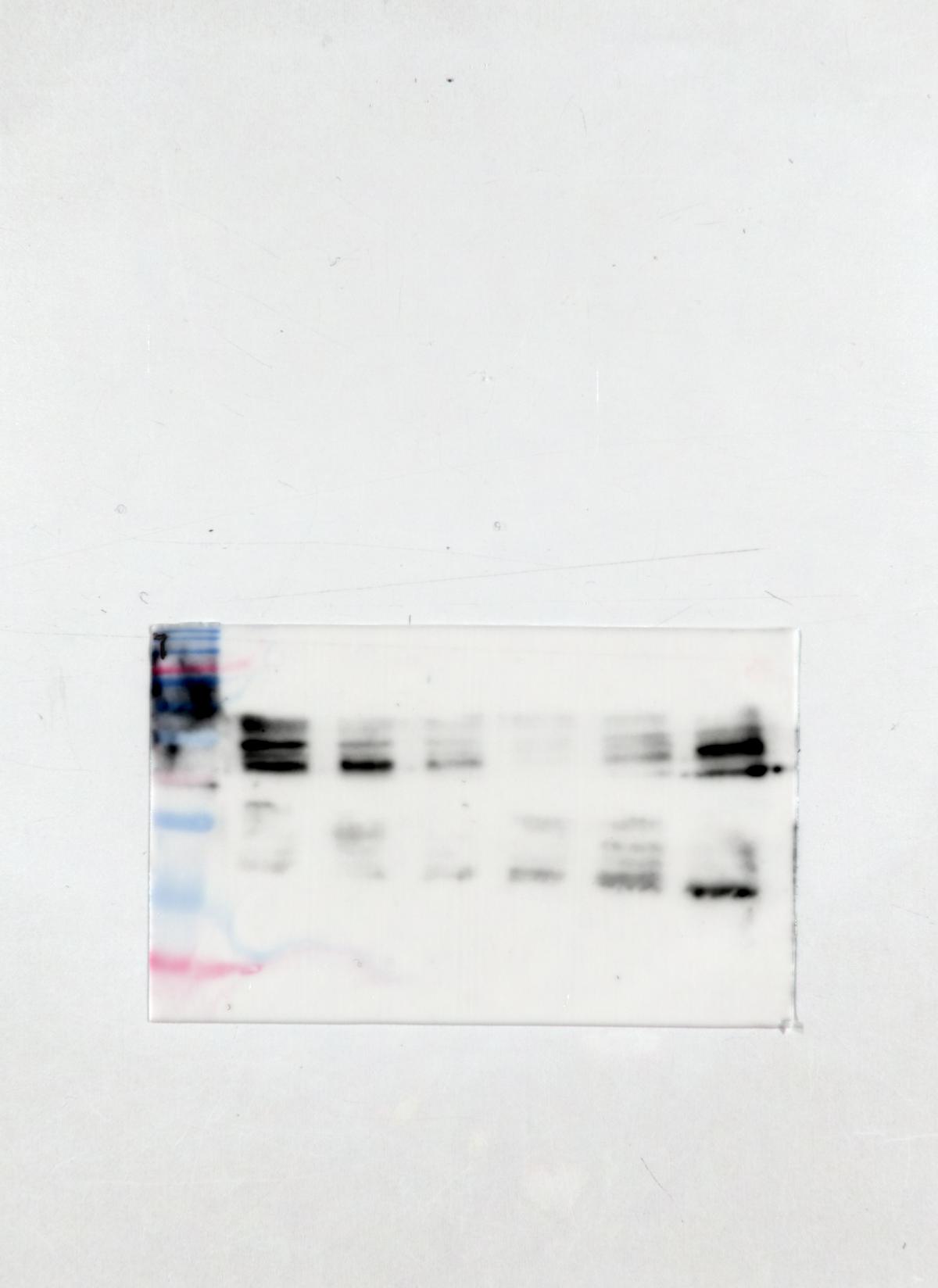

Supplement: Supplementary file 1 [file biomolecules-14-00901-s001.zip › Fig.5B Caspase 7 and cleaved Caspase 7.jpg]

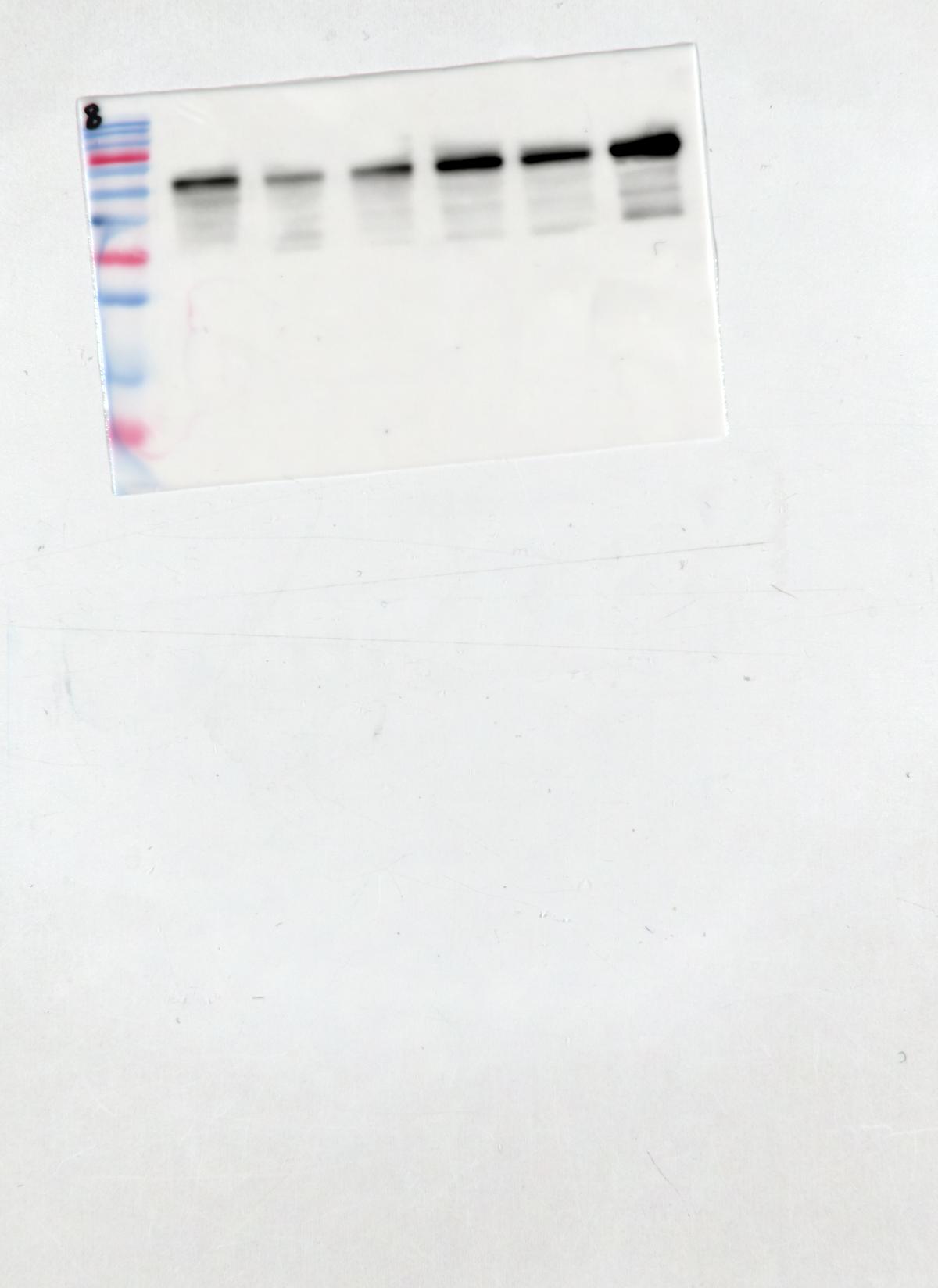

Supplement: Supplementary file 1 [file biomolecules-14-00901-s001.zip › Fig.5B Caspase 8.jpg]

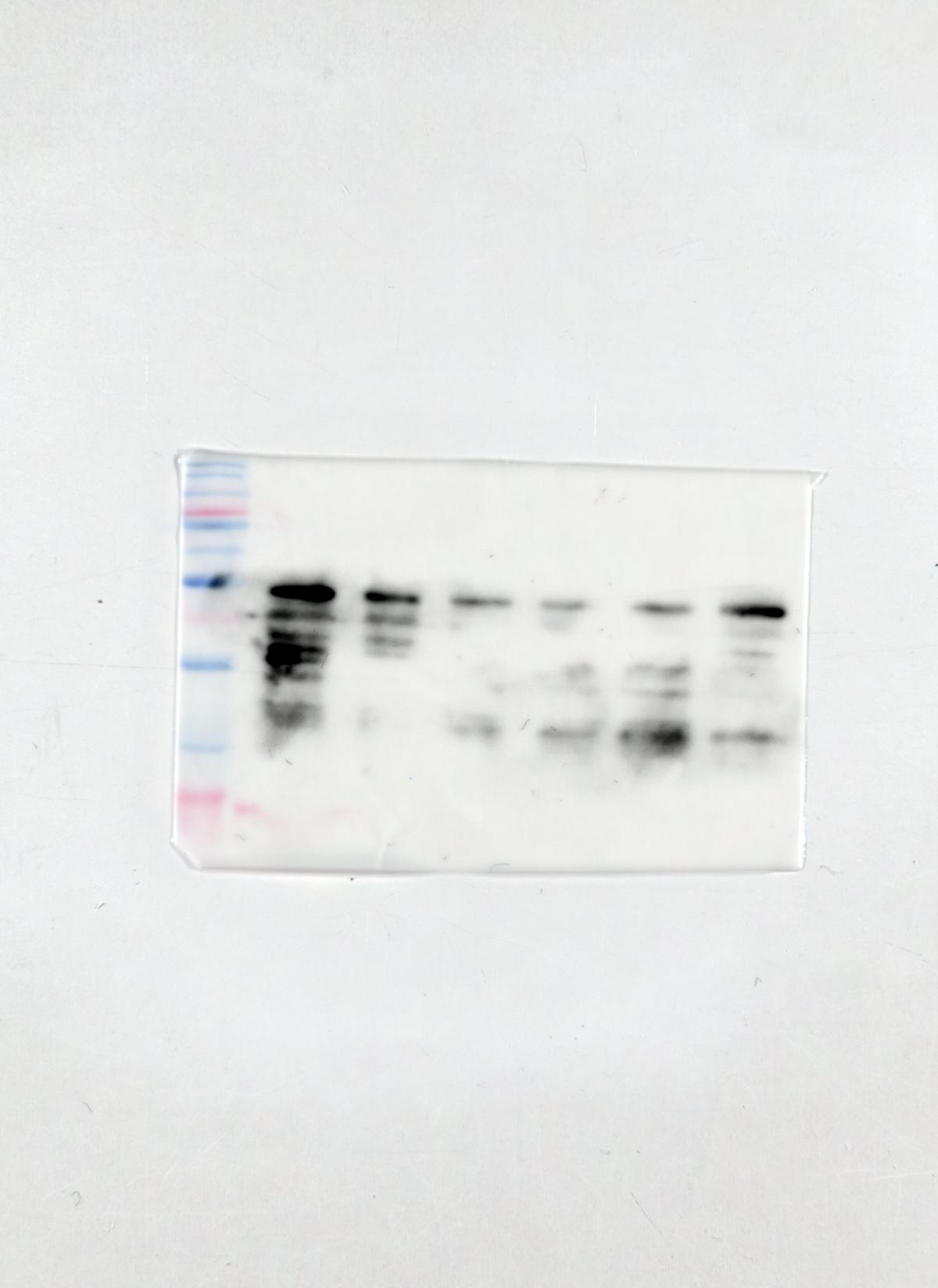

Supplement: Supplementary file 1 [file biomolecules-14-00901-s001.zip › Fig.5B Caspase3 and cleaved Caspase3.jpg]

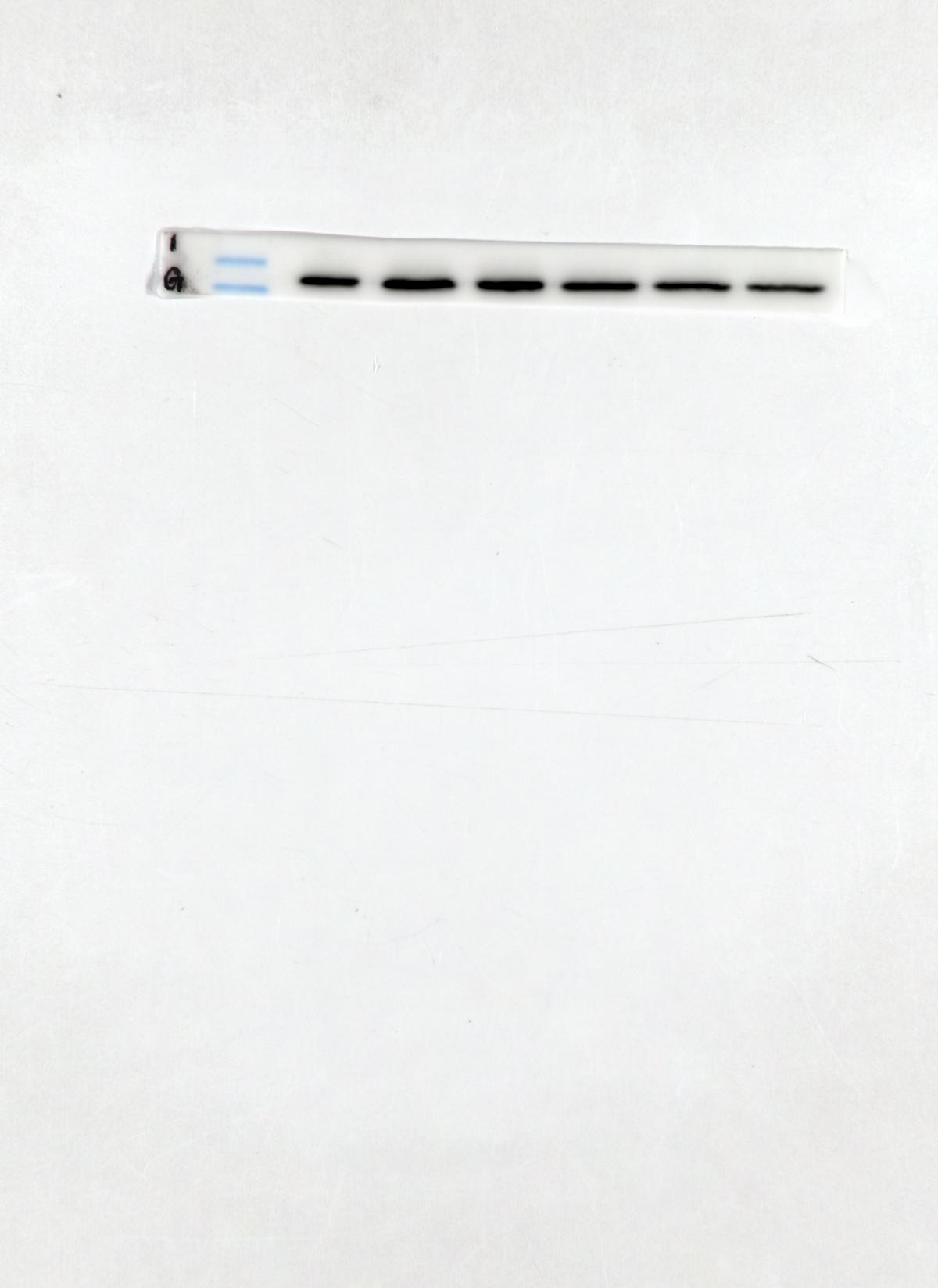

Supplement: Supplementary file 1 [file biomolecules-14-00901-s001.zip › Fig.5B GAPDH.jpg]

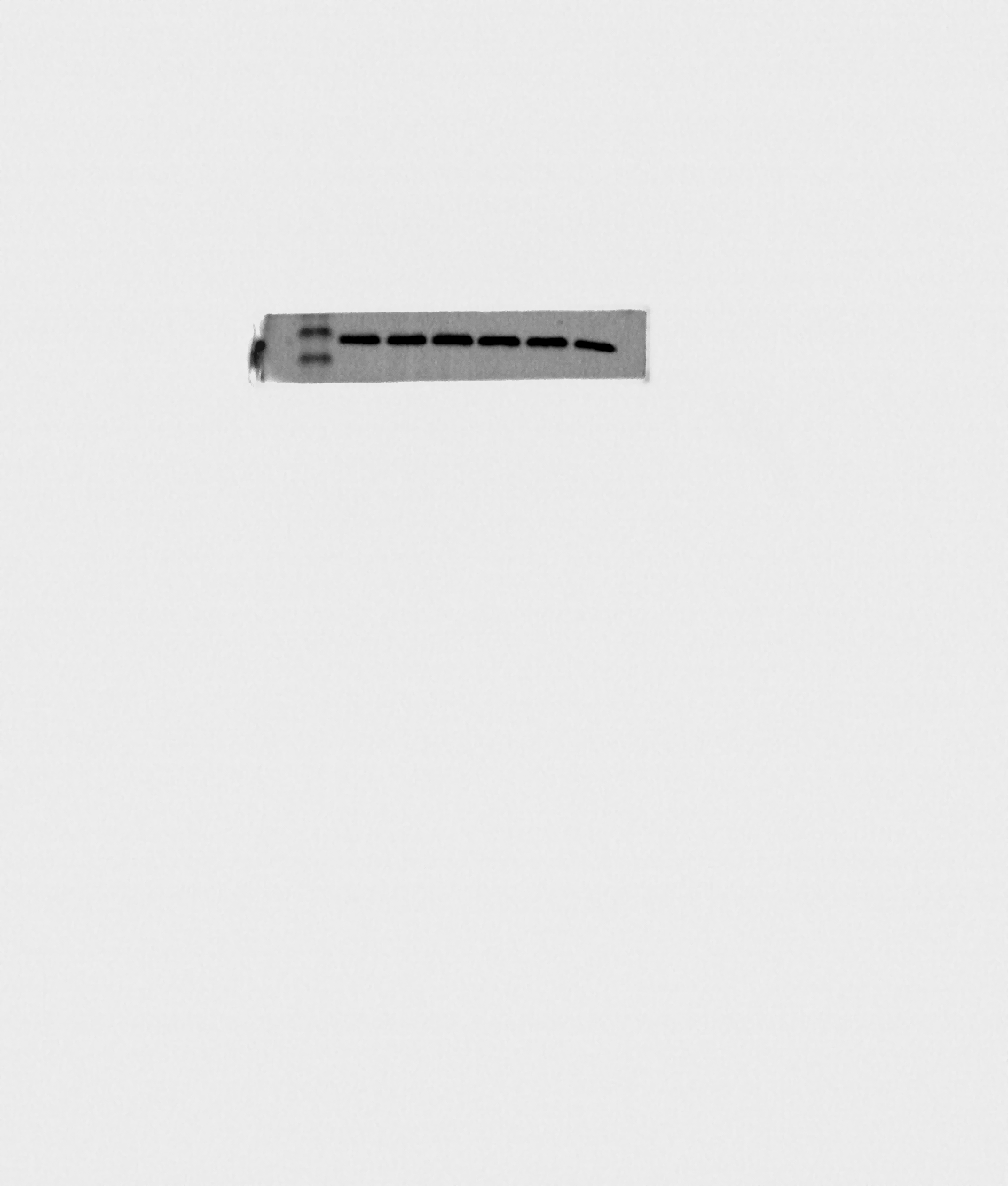

Supplement: Supplementary file 1 [file biomolecules-14-00901-s001.zip › Fig.5D ACTB.tif]

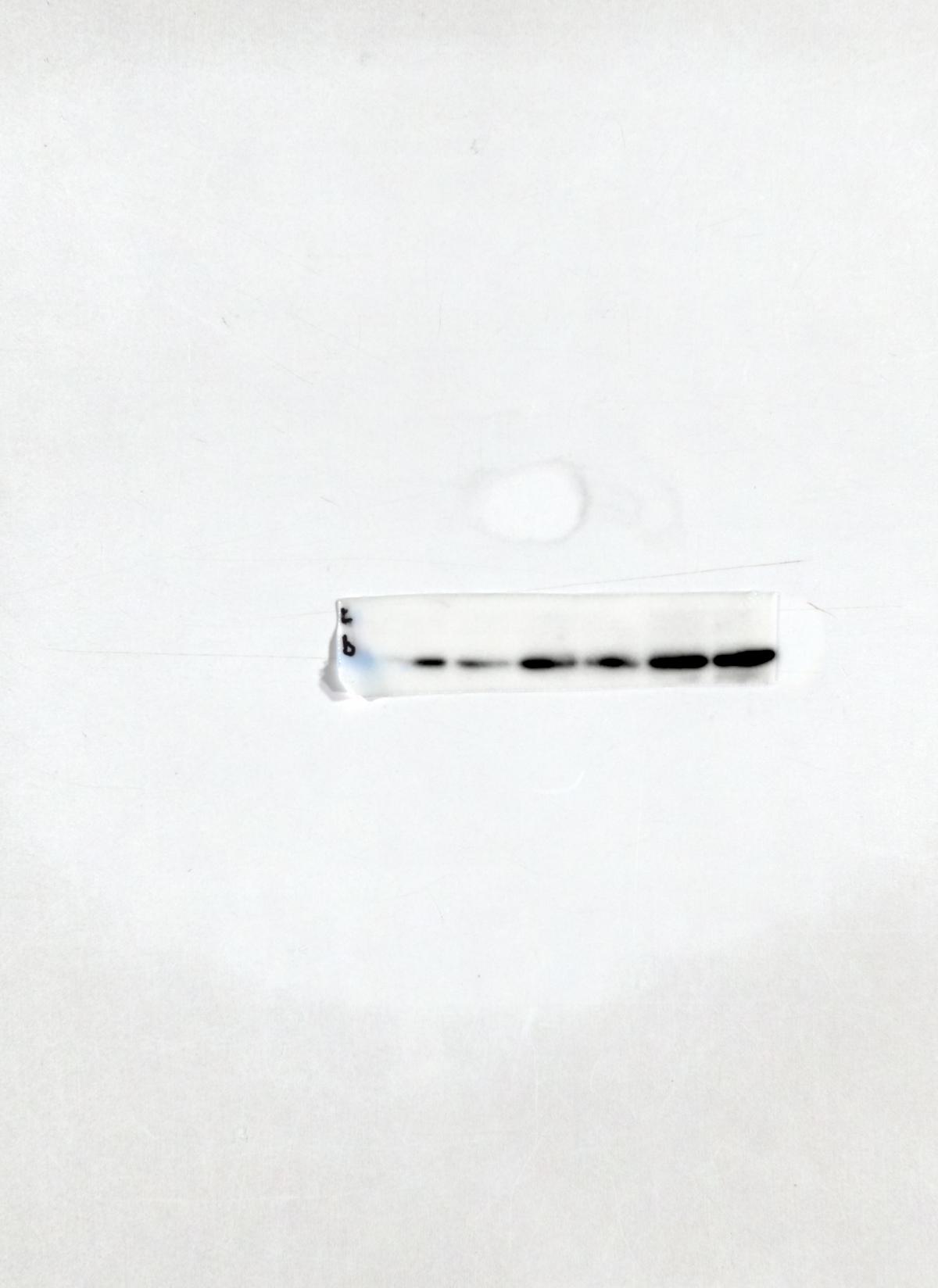

Supplement: Supplementary file 1 [file biomolecules-14-00901-s001.zip › Fig.5D Bax.jpg]

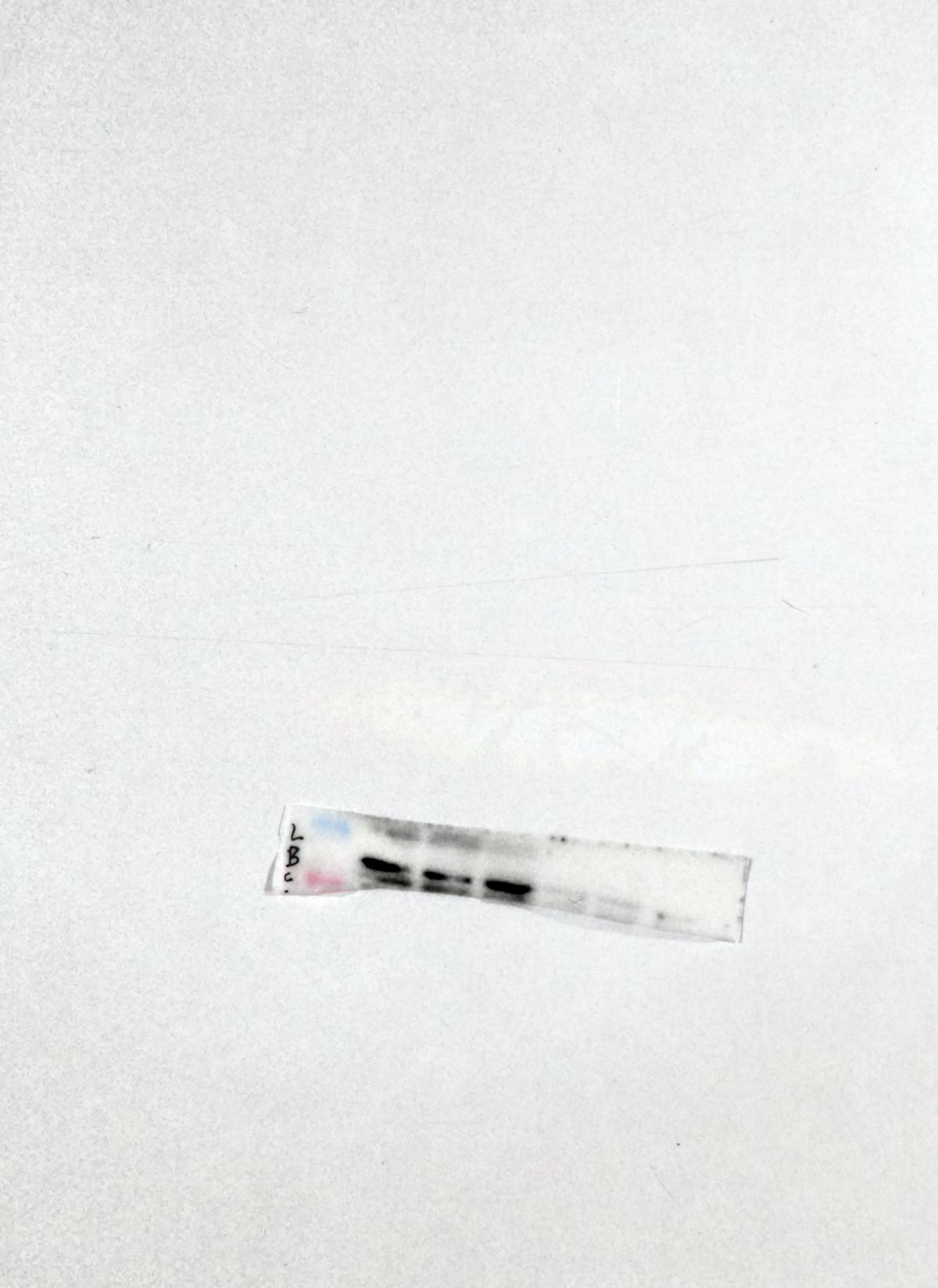

Supplement: Supplementary file 1 [file biomolecules-14-00901-s001.zip › Fig.5D Bcl-2.jpg]

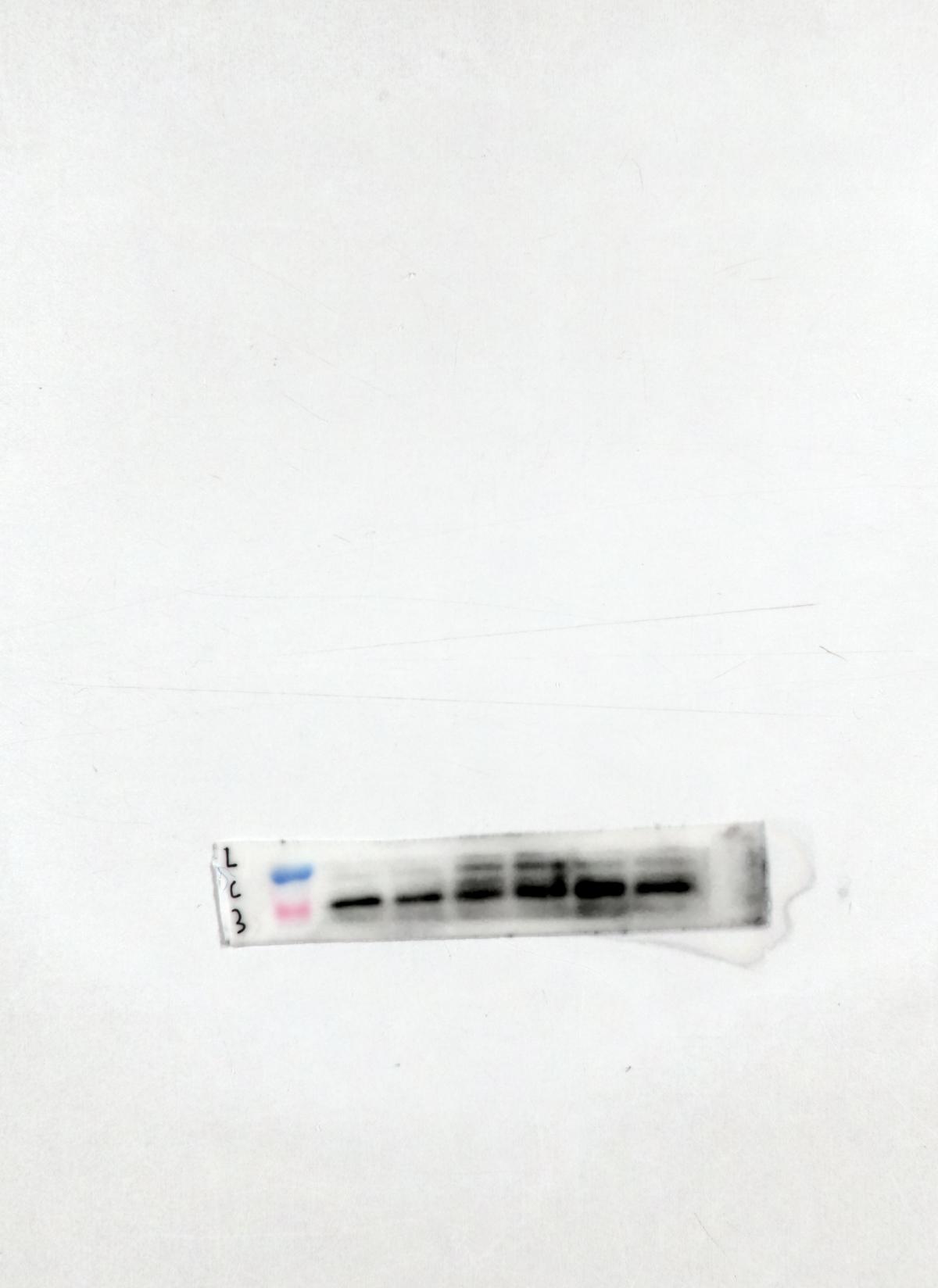

Supplement: Supplementary file 1 [file biomolecules-14-00901-s001.zip › Fig.5D Caspase 3.jpg]

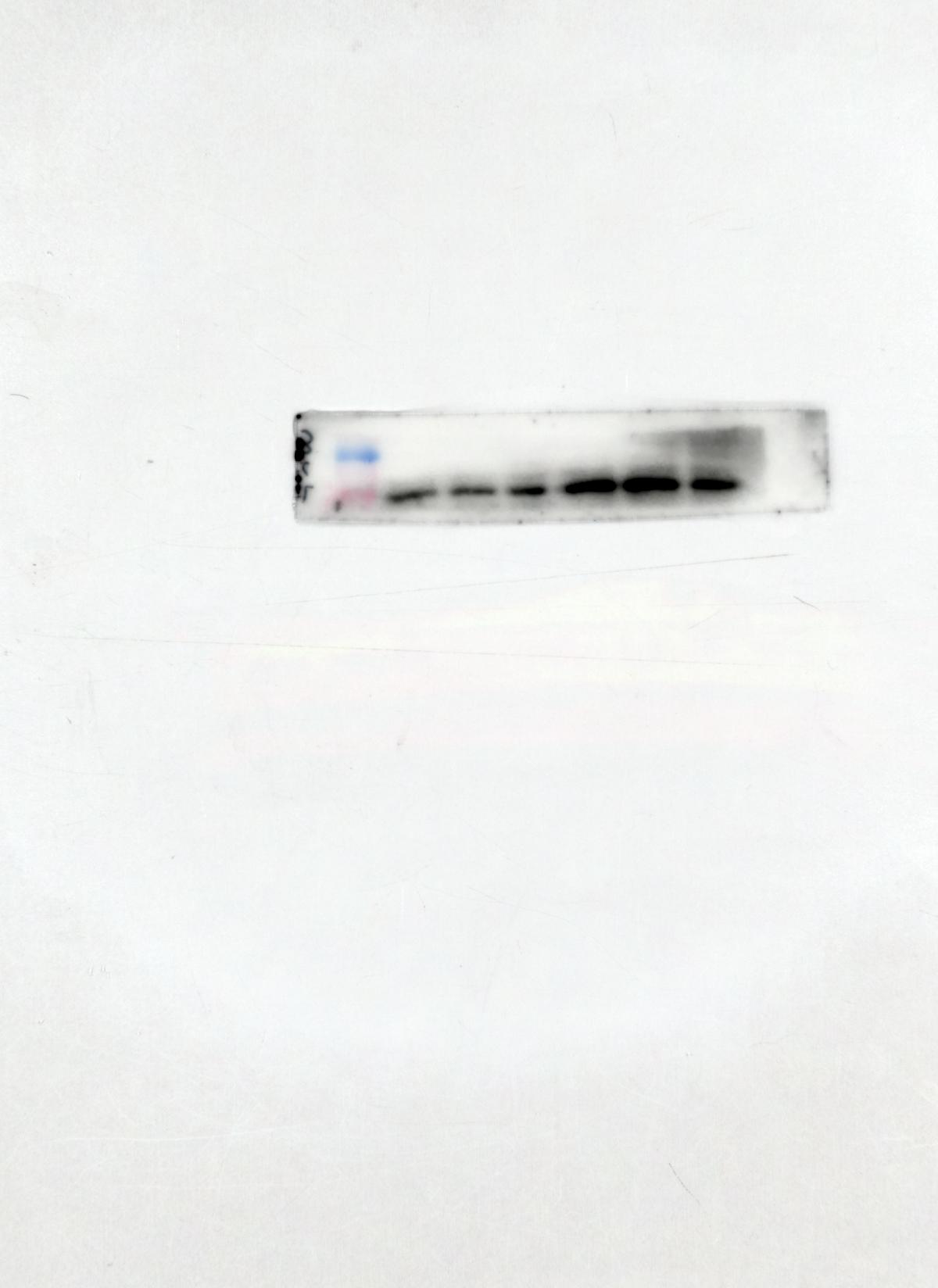

Supplement: Supplementary file 1 [file biomolecules-14-00901-s001.zip › Fig.5D Caspase 7.jpg]

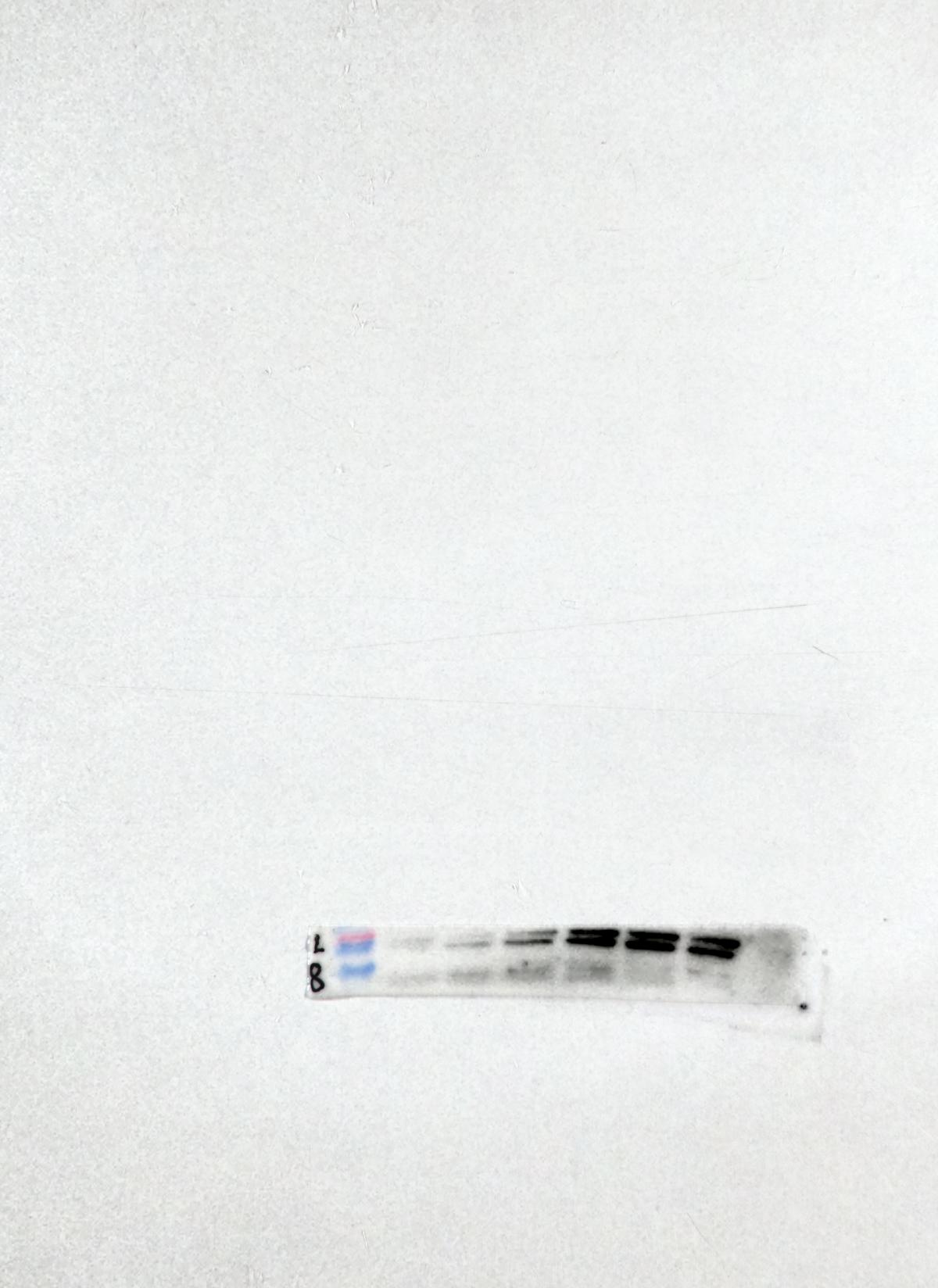

Supplement: Supplementary file 1 [file biomolecules-14-00901-s001.zip › Fig.5D Caspase-8.jpg]
